# Beneficial Effects of Bariatric Surgery-Induced by Weight Loss on the Proteome of Abdominal Subcutaneous Adipose Tissue

**DOI:** 10.3390/jcm9010213

**Published:** 2020-01-13

**Authors:** Bárbara María Varela-Rodríguez, Paula Juiz-Valiña, Luis Varela, Elena Outeiriño-Blanco, Susana Belén Bravo, María Jesús García-Brao, Enrique Mena, José Francisco Noguera, Javier Valero-Gasalla, Fernando Cordido, Susana Sangiao-Alvarellos

**Affiliations:** 1 Endocrine, Nutritional and Metabolic Diseases Group, Faculty of Health Sciences, Universidade da Coruña, Campus de Oza, 15006 A Coruña, Spain; barbara.varela.rodriguez@gmail.com (B.M.V.-R.); paula.juiz.valina@udc.es (P.J.-V.); fernando.cordido.carballido@sergas.es (F.C.); 2 INIBIC (Instituto de Investigación Biomédica de A Coruña), Xubias de Arriba, 84. 15006 A Coruña, Spain; 3 CICA (Centro de Investigaciones Científicas Avanzadas), As Carballeiras, s/n Campus de, San Vicente de Elviña, 15008 A Coruña, Spain; 4 Program in Integrative Cell Signaling and Neurobiology of Metabolism, Department of Comparative Medicine, Yale University School of Medicine, New Haven, CT 06520, USA; luis.varela@yale.edu; 5 Department of Endocrinology, Hospital Universitario A Coruña, A Coruña, 15006 A Coruña, Spain; Elena.Outeirino.Blanco@sergas.es; 6 Proteomic Unit, Health Research Institute of Santiago de Compostela (IDIS), Santiago de Compostela, 15705 A Coruña, Spain; sbbravo@gmail.com; 7 Department of Digestive and General Surgery, Hospital Universitario A Coruña, 15006 A Coruña, Spain; MA.Jesus.Garcia.Brao@sergas.es (M.J.G.-B.); Enrique.Mena.del.Rio@sergas.es (E.M.); jose.Francisco.Noguera.Aguilar@sergas.es (J.F.N.); 8 Department of Plastic, Reconstructive & Aesthetic Surgery. Hospital Universitario A Coruña, 15006 A Coruña, Spain; Javier.Valero.Gasalla@sergas.es

**Keywords:** bariatric surgery, proteome, abdominal adipose tissue, lipogenesis, mitochondria, metabolism, immune system

## Abstract

Bariatric surgery (BS) is the most effective treatment for obesity and has a positive impact on cardiometabolic risk and in the remission of type 2 diabetes. Following BS, the majority of fat mass is lost from the subcutaneous adipose tissue depot (SAT). However, the changes in this depot and functions and as well as its relative contribution to the beneficial effects of this surgery are still controversial. With the aim of studying altered proteins and molecular pathways in abdominal SAT (aSAT) after body weight normalization induced by BS, we carried out a proteomic approach sequential window acquisition of all theoretical mass spectra (SWATH-MS) analysis. These results were complemented by Western blot, electron microscopy and RT-qPCR. With all of the working tools mentioned, we confirmed that after BS, up-regulated proteins were associated with metabolism, the citric acid cycle and respiratory electron transport, triglyceride catabolism and metabolism, formation of ATP, pyruvate metabolism, glycolysis/gluconeogenesis and thermogenesis among others. In contrast, proteins with decreased values are part of the biological pathways related to the immune system. We also confirmed that obesity caused a significant decrease in mitochondrial density and coverage, which was corrected by BS. Together, these findings reveal specific molecular mechanisms, genes and proteins that improve adipose tissue function after BS characterized by lower inflammation, increased glucose uptake, higher insulin sensitivity, higher de novo lipogenesis, increased mitochondrial function and decreased adipocyte size.

## 1. Introduction

Persistent adipose tissue (AT) growth promotes obesity, a chronic disease [1] with extremely complex management, and which is widely recognized as the largest and fastest growing public health problem in the developed and developing world [2,3]. It is a multifactorial disease associated with high mortality and comorbid conditions such as type 2 diabetes (T2D), metabolic syndrome (MetS), hypertension, dyslipidemia, certain cancers, cellular senescence [4], sleep apnea and osteoarthritis [5,6], and has been found to be a risk factor for dementia and mild cognitive impairment [7]. However, obesity is not always synonymous with disease. The “expandability hypothesis” suggests that it is not the absolute amount of AT, but it is just this fat accumulation in organs outside of the AT that affects metabolic homeostasis [8]. Broadly speaking, humans’ AT can be divided into subcutaneous AT (SAT) and visceral AT (VAT). SAT represents over 80% of total body fat and can be classified as central (abdominal) or extremity compartments (gluteal-femoral), each of which has different functional characteristics [9]. Many studies have suggested that this distribution is strongly associated with insulin resistance, with VAT being the strongest predictor of insulin resistance or cardiometabolic risk [10,11]. It has been stated that subcutaneous fat has a neutral role in insulin sensitivity, as the surgical removal of large amounts of SAT (> 10 kg) by liposuction in obese humans does not cause improvement in insulin resistance or other metabolic benefits [12]. Some authors have even claimed that SAT has a protective role against insulin resistance, something that would be related to adiponectin levels [13]. However, recent studies have shown that abdominal SAT (aSAT), but not gluteal-femoral fat is also related to insulin resistance, observing that abdominal fat, regardless of whether it has a visceral or subcutaneous location, is negatively correlated with adiponectin levels [14]. In 2017 Vegiopoulos et al. showed that unlike obese patients who have insulin sensitivity, those with insulin resistance have exceeded storage capacity, decreased adipocyte number, enhanced adipocyte size, higher inflammation and immune cell infiltration, adverse adipokine profile and higher fibrosis [15]. However, the relative contribution of aSAT to insulin resistance and metabolic disorders is still controversial.

Bariatric surgery is the most effective treatment for obesity and it has a positive impact on cardiometabolic risk and in the remission of T2D in obese patients. The normalization of glucose metabolism that often occurs before weight loss and its magnitude is far greater than that explained by the loss of weight alone, although the mechanisms involved in this improvement have not been fully clarified [16]. However, different studies have shown that reductions in waist circumference after bariatric surgery were associated with a greater probability of T2D remission [17,18,19]. If we also take into account that after BS, the immense majority of total fat mass is lost from SAT [20], it is logical to think that the changes in mass and SAT function could contribute to the beneficial effects of bariatric surgery. Based on the above, and in order to have a wider view of all the changes that occur in aSAT after weight normalization induced by BS, we carried out a proteomic approach to identify the pathways altered. For this purpose, qualitative and quantitative LC–MS/MS analysis of paired biopsies was performed to identify and quantify proteins. In order to validate some results obtained with proteomic analysis in healthy control patients and obese patients before and after BS, we quantified, the protein expression levels of the lipogenic and insulin signaling enzymes using Western blot. We also analyzed mRNA expression levels of genes related to inflammation and metabolism as well as mitochondrial parameters (such as density, coverage or area), and adipocyte size distribution. 

## 2. Materials and Methods

### 2.1. Patients and Sample Collection

This study was approved by the Research Ethics Committee of Galicia, Spain (reference 2014/135); written informed consent was obtained from all patients.

To carry out this study we collected aSAT samples from three groups of patients: morbidly obese patients who underwent bariatric surgery (Roux-en-Y gastric bypass (RYGB) or sleeve gastrectomy (SG)), some of these same patients after normalizing their body weight (when the body mass index (BMI) was less than 30 kg/m^2^) and undergoing abdominoplasty, and healthy non-obese patients undergoing abdominal hernia surgeries. All the samples were obtained during the surgeries, without altering the protocol of the same ones. The fat biopsies were divided into three pieces. One of them was placed in dry ice immediately after the extraction and stored at –80 °C until analysis. The second piece of the aSAT was paraffin-embedded, and the third piece was processed for electron microscopy analysis at a later stage.

### 2.2. Protein Identification by LC–MS/MS

Total extracts were prepared in lysis buffer (pH 7.5) containing 50 mM Trizma-HCl, 1 mM EGTA, 1 mM EDTA, 50 mM NaF, 5 mM sodium pyrophosphate, 1 mM sodium orthovanadate, 1% Triton X-100, protease inhibitor cocktail (complete Mini EDTA-free Protease Inhibitor Cocktail; Roche Diagnostics), 0.25 M sucrose and 0.1% β-mercaptoethanol. Tissue lysates were centrifuged at 12,000× *g* for 30 min at 4 °C. In order to perform protein identification and quantification, an equal amount of protein (100 μg) from four patients before and after BS, were boiled in SDS-loading buffer (62.5 mM Trizma-HCl pH 6.8, 5% SDS, 10% glycerol, 5% β-mercaptoethanol, 0.0025% bromophenol blue) during 5 min and then were loaded on a 10% sodium dodecyl sulphate polyacrylamide gel electrophoresis (SDS-PAGE) and stopped when the front had penetrated 3 mm into the resolving gel [21,22]. The protein band was stained using Sypro-Ruby (Lonza, Switzerland), excised, and processed for in-gel, manual tryptic digestion as described elsewhere [23]. Peptides were extracted by carrying out three 20-min incubations in 40 μL of 60% acetonitrile dissolved in 0.5% HCOOH. The resulting peptide extracts were pooled, concentrated in a SpeedVac, and stored at –20 °C.

#### 2.2.1. Mass Spectrometric Analysis Using a Shotgun Data-Dependent Acquisition (DDA) Method

A total of 4 μL (4 μg) of digested peptides were separated by Reverse Phase Chromatography using a micro liquid chromatography system (Eksigent Technologies nanoLC 400, AB SCIEX, Madrid, Spain) coupled to a high-speed TripleTOF 6600 mass spectrometer (AB SCIEX, Madrid, Spain) with a microflow source. The analytical column was a silica-based reversed phase column Chrom XP C18 150 mm × 0.30 mm, 3 mm particle size and 120 Å pore size (Eksigent, AB SCIEX, Madrid, Spain). The trap column was a YMC-TRIART C18 (YMC Europe GmbH, Dinslaken, Germany) with a 3 mm particle size and 120 Å pore size, switched online with the analytical column. The loading pump works at 10 μL/min using a solution of 0.1% formic acid in water. The gradient pump works at 5 μL/min under gradient elution conditions, using 0.1% formic acid in water as the mobile phase A and 0.1% formic acid in acetonitrile as the mobile phase B. Peptides were separated using a 90 min gradient ranging from 2% to 90% of mobile phase B. 

Data acquisition was performed with a TripleTOF 6600 System, ASSY. Nº 5060890) (AB SCIEX, Madrid, Spain) using a data-dependent workflow. The conditions in the source and interface were: ionspray voltage floating (ISVF) 5500 V, curtain gas (CUR) 25, collision energy (CE) 10 and an ion source gas 1 (GS1) 25. The instrument was operated with an Analyst TF 1.7.1 software (AB SCIEX, Madrid, Spain). The switching criteria were set to ions greater than mass to charge ratio (m/z) 350 and smaller than m/z 1400 with a charge state of 2–5, mass tolerance 250 ppm and an abundance threshold of more than 200 counts (cps). Former target ions were excluded for 15 s. The instrument was automatically calibrated every four hours using with external calibrant tryptic peptides from PepCalMix.

##### Data Analysis

Data files from the MS/MS analysis were processed using the ProteinPilotTM version 5.0.1 software (AB SCIEX, Madrid, Spain), which uses the algorithm ParagonTM for the database search and ProgroupTM for data grouping. Data were searched using a human-specific Uniprot database specifying iodoacetamide as Cys alkylation and methionine oxidation as a fixed modification. The false discovery rate was performed using a non-linear fitting method displaying only those results that reported a 1% global false discovery rate or better [24].

#### 2.2.2. Label-Free Quantitative Analysis Sequential Window Acquisition of all Theoretical Mass Spectra (SWATH-MS)

##### Creation of the Spectral Library 

In order to build the MS/MS spectral libraries, the peptide solutions were analyzed by a shotgun DDA approach using micro-LC–MS/MS. To obtain a good representation of the peptides and proteins present in all the samples, pooled vials of samples from each group were prepared using equal mixtures of the original samples. Four microliters of each pool were separated and analyzed using the same conditions as before with minimal modifications. The gradient run consisted of 5%–95% B for 30 min, 5 min at 90% B, and finally five min at 5% B for column equilibration, for a total run time of 40 min. In the mass spectrometry, the acquisition mode consisted of a 250 ms survey MS scan from 400 to 1250 m/z followed by an MS/MS scan from 100 to 1500 m/z (25 ms acquisition time) of the top 65 precursor ions from the survey scan, for a total cycle time of 2.8 s. The fragmented precursors were then added to a dynamic exclusion list for 15 s and any single-charged ions were excluded from the MS/MS analysis.

The peptide and protein identifications were performed using the Protein Pilot software (version 5.0.1, AB Sciex, Madrid, Spain) using a human-specific Uniprot database, specifying iodoacetamide as cys alkylation and methionine oxidation as the fixed modification. For both peptides and proteins, we fixed the false discovery rate (FDR) at 1. The MS/MS spectra of the identified peptides were then used to generate the spectral library for the sequential window acquisition of all theoretical (SWATH) peak extraction using the add-in for the PeakView Software (version 2.2, Sciex) MS/MSALL with the SWATH Acquisition MicroApp (version 2.0, Sciex). Only peptides with a confidence score above 99% were included in the spectral library.

##### Relative Quantification by SWATH Acquisition 

Eight samples (four samples from patients with morbid obesity and another four from these same patients after undergoing bariatric surgery and normalizing their body weight, BMI < 30) were analyzed using data-independent acquisition (DIA) method. Each sample (4 μL) was analyzed using the LC–MS/MS equipment and LC gradient described above for building the spectral library but instead using the SWATH-MS acquisition method. The method consisted of repeating a cycle of 65 time-of-flight (TOF) MS/MS scans (400–1500 m/z, high-sensitivity mode, 50 ms acquisition time) of overlapping sequential precursor isolation windows of the variable width (1 m/z overlap) covering the 400–1250 m/z mass range with a previous TOF MS scan (400–1250 m/z, 50 ms acquisition time) for each cycle. Total cycle time was 6.3 s. For each sample set, the width of the 65 variable windows was optimized according to the ion density found in the DDA runs using a SWATH variable window calculator worksheet from Sciex.

##### Data Analysis 

The targeted data extraction of the fragment ion chromatogram traces from the SWATH runs was performed by PeakView (version 2.2) using the SWATH Acquisition MicroApp (version 2.0). This application processed the data using the spectral library created from the DDA data. We selected based on signal intensity, ten peptides per protein and seven fragments per peptide, where any modified peptides were excluded from the processing. We used five-minute windows and 30 ppm widths to extract the ion chromatograms. After the retention times were adjusted for all peptides selected, the integrated peak areas were directly exported to the MarkerView 1.3.1 software, AB SCIEX, for relative quantitative analysis. Data alignment by MarkerView compensates for minor variations in both mass and retention time values, ensuring that identical compounds in different samples are accurately compared to one another. To control for possible uneven sample loss across the different samples during the sample preparation process, we performed a global normalization based on the total sum of all the peak areas extracted from all the peptides and transitions across the replicates of each sample. Unsupervised multivariate statistical analysis using principal component analysis (PCA) was performed to compare the data across the samples, using Pareto scaling (data not shown). The average MS peak area of each protein was derived from the replicates of the SWATH-MS of each sample followed by a Student’s *t*-test analysis using the MarkerView software for comparison among the samples based on the averaged area sums of all the transitions derived for each protein. The *t*-test indicates how well each variable distinguishes the two groups, reported as a *p*-value. For each library, its set of differentially expressed proteins (*p*-value *<* 0.5) with a 1.5 fold increase or decrease was selected.

#### 2.2.3. Gene Ontology Analysis, Functional Networks and Pathway Mapping

Enriched gene ontology (GO) categories, protein classes and biological pathways within the differential body weight-expressed protein datasets (qualitative analysis, i.e., proteins identified only during obesity or after normalization of body weight, but without quantification) were performed using the PANTHER enrichment analysis tool (version 14.1, www.pantherdb.org/) [25].

The GO analysis for the quantitative analysis, namely of proteins, up-regulated and down-regulated following treatment with bariatric surgery were initially performed using the FunRich stand-alone enrichment analysis tool FunRich (version 3.1.3, http://www.funrich.org/) [26]. Reactome biological pathway enrichment analysis of deregulated proteins by weight loss after BS was performed with the STRING tool (version 11, https://string-db.org/) [27]. In both cases, we only used proteins that presented statistically significant differences. Using these proteins, we also performed a heatmap. The expression-based heatmap was plotted using matrix2png, a freely available web server (version 1.2.2, http://www.chibi.ubc.ca/matrix2png/).

### 2.3. Western Blot

Western blot analysis to estimate fatty acid synthase (FAS), serine/threonine kinase 1 (AKT), phospho-AKT (pAKT), acetyl-CoA carboxylase (ACC), phospho-ACC (pACC), protein kinase AMP-activated catalytic subunit alpha 1 (AMPKα1), phospho-AMPKα (pAMPKα) and glyceraldehyde 3-phosphate dehydrogenase (GAPDH) protein expression levels was completed for aSAT. The total extract were prepared as described previously and 15 µg of total protein lysates was boiled in SDS-loading buffer during 5 min and then fractionated with SDS-PAGE (7% or 10% gel), electrotransferred on a polyvinylidene fluoride membrane (Millipore). After blocking with 5% BSA in 1 × Tween-Tris-buffered saline (TTBS; 0.05% Tween, 25 mM Tris-HCl, pH 7.5 and 150 mM NaCl) for 1 h at room temperature, membranes were washed three times with TBST, then incubated overnight at 4 °C with the primary antibody: ACC, phospho-ACC-Ser79 (pACC; Millipore); AMPKα1, phospho-AMPKα-Thr172 (pAMPKα), phospho-AKT-Ser473 (pAKT), AKT (Cell Signaling); FAS (Santa Cruz Biotechnology) and GAPDH (Thermo Fisher Scientific). For protein detection, the membranes were washed three times with TBST and then were incubated with horseradish peroxidase-conjugated secondary antibodies (Dako) for 1 h at room temperature. The targeted protein was revealed by enhanced chemiluminescence (Pierce ECL, ThermoFisher) and blots were imaged by detecting chemiluminescence on an Amersham Imager 600 (Ge Healthcare Life Sciences). The intensity of each band was quantified by densitometry using the ImageJ Software (Version 1.52a, National Institutes of Health, USA) [28]. The protein levels were normalized to GAPDH in each sample.

### 2.4. Hexokinase (HK) Activity

The aSAT samples were homogenized in four volumes ice-cold buffer: 20 mM Tris-HCl, (pH 7.4), 250 mM sucrose, 1 mM EDTA, 1mM dithiothreitol, 100 mM NaF and protease inhibitor cocktail (Roche, Stockholm, Sweden). Enzyme activities were determined by NADH formation, following absorbance at 340 nm, using a microplate reader (Tecan, Sunrise). The reactions were started by the addition of homogenates (50 µL) and substrates (20 µL, omitted in controls) to the reaction mixture (final volume 0.25 mL) and allowing the reactions to proceed at 37 °C for five min. HK activity was measured using methods previously described with slight modifications [29]. The activity levels were normalized to the total protein of each sample. The protein concentration was determined using the Bradford method (Biorad, Bradford Protein Assay Kit), using bovine serum albumin as a standard.

### 2.5. Quantitative Real-Time PCR

Total RNA was extracted from AT using TRIzol reagent (Invitrogen). RNA integrity and quality/concentrations were determined by agarose gel electrophoresis and spectrophotometry in an ND-1000 NANODROP 385 spectrophotometer (Thermo-Scientific), respectively. Real-time PCR was performed on a Roche LightCycler 480 Real-Time PCR Detection System. For mRNA quantification, 0.8 μg of total RNA per sample were retro-transcribed (RT) in a 20 μL reaction, using SuperScript IV reverse transcriptase and random hexamers (Invitrogen). For PCR, we used SYBR Green qPCR Master Mix (Roche). The primers used were: *F-box and leucine-rich repeat protein-10* (*FBXL10*; NM_032590.4) forward 5′: TACGACGAGAACGAGGACTT and reverse 5′: AGGCATCTTAATTCCCAGTCCA; *Importin 8* (*IPO8*; NM_006390.3) forward 5´: ACAATGTGTCTCCGTGCCAT and reverse 5´: AGCTTGCACTGCTCTGTGAT; *Alcohol dehydrogenase 1B (class I), beta polypeptide* (*ADH1B*; NM_001286650.1) forward 5´: AAGGGGGCTGTTTATGGTGG and reverse 5´: ACGTCAGGACGGTACGGATA; *FAS* (AY451392.1) forward 5´: CTGCACTTCCATAGCCCCAA *and* reverse 5´: AAGGAGTTGATGCCCACGTT; *Solute carrier family 2 member 4* (*SLC2A4*, also known as *GLUT4*; M20747.1) Forward 5´: CGTCGGGCTTCCAACAGATA and reverse 5´: CACCTTCTGAGGGGCATTGA and *S100 calcium binding protein A8* (*S100A8*; NM_001319201.1) forward 5´: TGTTGACCGAGCTGGAGAAA and reverse 5´: CCCTGTAGACGGCATGGAAAT. For data analysis, relative standard curves were constructed from serial dilutions of one reference sample cDNA, and the input value of the target gene was standardized to the geometric mean of the two control genes, *IPO8* and *FBXL10*, for each sample. PCR was initiated by one hold of 95 °C for 10 min, followed by 40 cycles of 15 s at 95 °C, 55 s at 60 °C and 5 s at 72 °C, followed by one hold of 72 °C for 10 min.

### 2.6. Electronic Microscopy and Mitochondrial Morphometrics

AT biopsies were fixed by immersion in 0.5% glutaraldehyde, 4% paraformaldehyde and 15% picric acid in 0.1 M phosphate buffer (PB, pH 7.4) during 48 hours at 4 °C. Samples were stored at 4 °C in PB + 0.1% sodium azide until processing. After post-fixation, samples were washed three times with PB and sectioned using a vibratome (50 μm). Sections were then osmicated (15 min in 1% osmium tetroxide) and dehydrated in increasing ethanol concentrations. During the dehydration, 1% uranyl acetate was added to the 70% ethanol to enhance ultrastructural membrane contrast. Then the samples were flat-embedded in Durcupan followed dehydration. Ultrathin sections were cut on a Leica ultra-microtome, collected on Formvar-coated single-slot grids, and analyzed with a Tecnai 12 Biotwin electron microscope (FEI) with an AMT XR-16 camera. Sections with distinguishable cells were analyzed by electron microscopy. Mitochondria coverage, density and aspect ratio were calculated using ImageJ. Mitochondrial density was estimated by dividing the number of mitochondrial profiles by the cytosolic area. Mitochondrial coverage was estimated by dividing the total area of mitochondria by the cytosolic areas. 

### 2.7. Adipocyte Size 

AT biopsies were excised and fixed in 4% paraformaldehyde for 24 h, dehydrated and embedded in paraffin. Paraffin blocks were sectioned at 5 μm, deparaffinized and rehydrated. Sections were stained, as described in [30] with hematoxylin and eosin (H&E) (Mayer’s hematoxylin and 1% eosin Y solution). The slides were dehydrated and cleared in xylene. Finally, they were mounted with resinous mounting medium and then to determine adipocyte size, pictures were taken with a light microscope (Olympus BX61). Adipocyte size was measured using the CellSens Dimension software (Olympus). 

### 2.8. Statistical Analysis

The proteomics data analysis was carried out, as explained above. The rest of the data were analyzed using SigmaStat 3.1 (Systat Software, Inc., Erkrath, Germany) and are presented as the means ± standard error means (SEM). Statistical significance was determined by one-way ANOVA with post hoc Tukey’s test (data with normal distribution) or Kruskal–Wallis test with post hoc Dunn’s test (data without normal distribution). *p* < 0.05 was considered significant. Different letters above bars indicate statistical significance. 

## 3. Results

### 3.1. Qualitative Analysis of Expressed Proteins in the aSAT before or after Weight Loss

**Figure 1:**
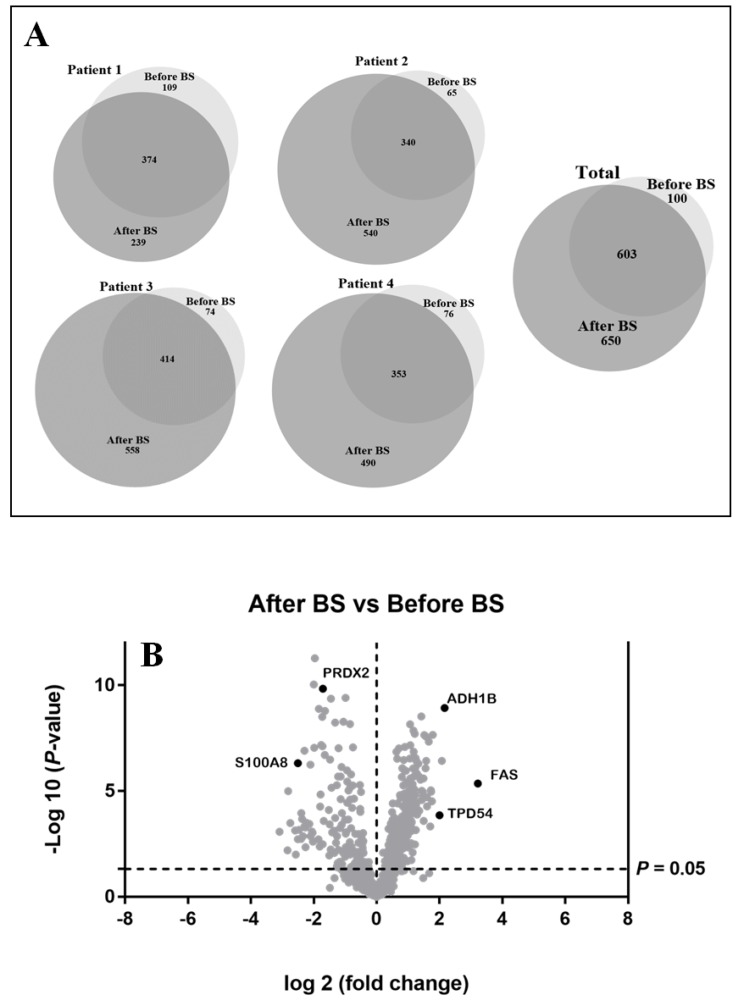
(**A**). Effect of obesity and bariatric surgery on protein expression in abdominal subcutaneous adipose tissue depot (aSAT). Venn diagrams showing the qualitative proteomic analysis of aSAT from four obese patients before bariatric surgery (before BS) and the same patients after body weight loss induced by bariatric surgery (after BS). They show the number of unique and overlapping proteins identified individually for each patient before and after bariatric surgery (Patient 1–4), as well as the total of proteins identified. (**B**). Graphical representation of quantitative proteomics data. Proteins are ranked in a volcano plot according to their statistical *p*-value (*y*-axis) and their relative abundance ratio (log2-fold change) between aSAT samples after BS and before BS (*x*-axis). Off-centered spots are those that vary the most between both groups. The cut-offs for significant changes are fold-changes of 1.5 and *p* < 0.05.

aSAT samples from the same patient were compared before and after weight loss to obtain an expressed-protein profile. Anthropometric and biochemical parameters of these patients are shown in Table 1. These data were taken from the patients’ medical history. The results for qualitative analysis are shown in Figure 1A. A total of 1353 proteins were identified. One-hundred proteins were expressed exclusively during obesity and 650 proteins after weight normalization. Six hundred and three proteins were common to both states. For all patients, it could be observed that the number of identified proteins increases after bariatric surgery (see Figure 1A), demonstrating that obesity presents lower protein variability at aSAT level.

The PANTHER classification system [25] was implemented on proteins identified only during obesity or after weight loss. The comparison between the 100 proteins expressed only during obesity and the 650 proteins expressed after bariatric surgery provided interesting evidence about the molecular effects induced by obesity and weight loss induced by bariatric surgery treatment. Table S1 shows the enriched GO categories for each of the three GO terms: cellular compartment, molecular function and biological process. The biological pathway and protein class analysis are also detailed in Table S1. We found that the percentage of proteins associated with the extracellular region and cell junction decreased after weight loss. On the contrary, the percentage of proteins that were part of organelles increased after bariatric surgery. After weight loss, there was a decrease in the proteins associated with the immune system process and response to a stimulus. Finally, it should be noted that many of the proteins that are only expressed after weight loss were associated with different metabolic pathways (see Table S1 for more details).

### 3.2. Differentially Expressed Proteins Quantified by SWATH-MS Analysis in the aSAT, before and after Weight Loss. Bioinformatic Analysis for Enriched Terms

**Figure 2:**
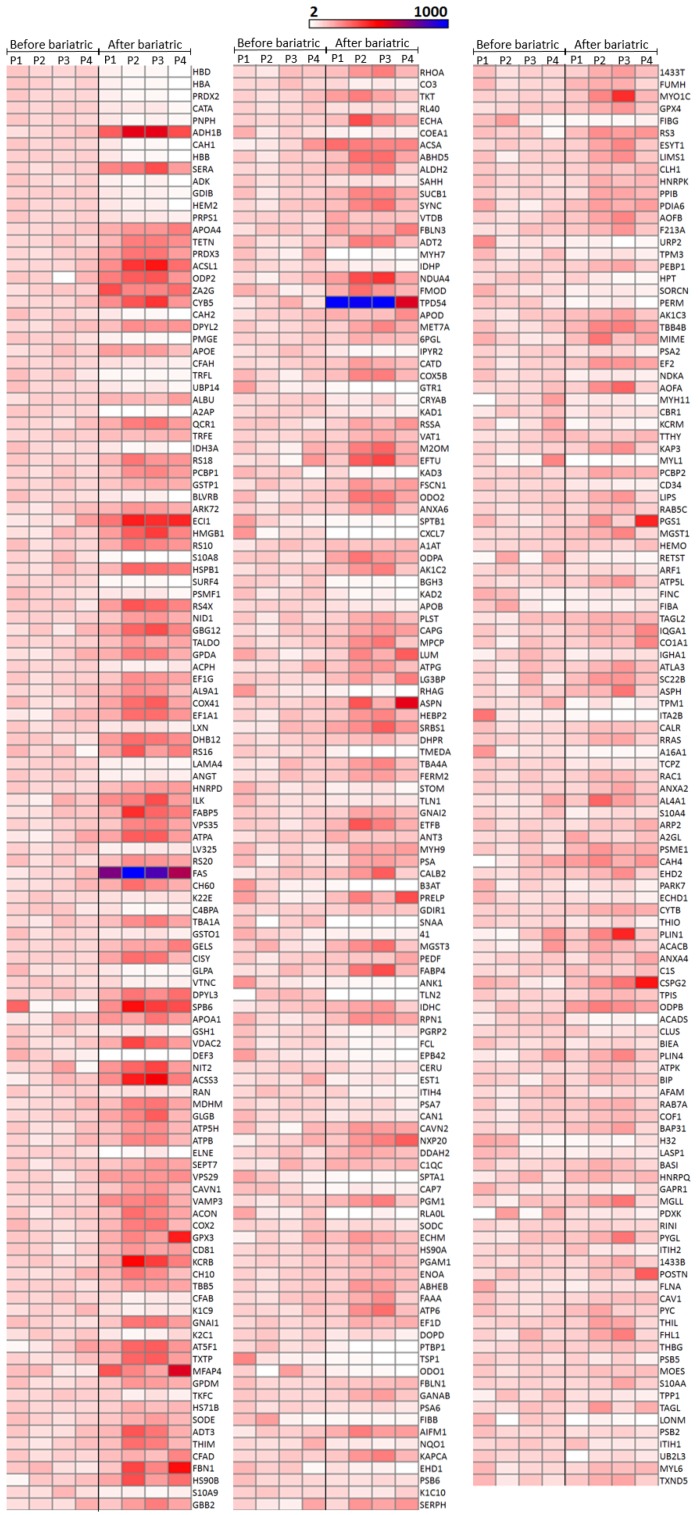
Heatmap of significantly regulated proteins by weight loss induced by bariatric surgery on aSAT using a sequential window acquisition of all theoretical mass spectra (SWATH-MS) technique. Effects of weight loss induced by bariatric surgery on aSAT protein expression. Three-hundred and sixty-four proteins were significantly regulated, 136 down-regulated and 228 up-regulated. The expression intensity of each protein varies from white to blue. The expression is given in arbitrary units and in order to adequately visualize the changes due to bariatric surgery, the expression value of each protein was normalized taking into account the mean of that protein for the four patients before undergoing bariatric surgery. This value was assigned as 100% and all values represented here were expressed based on that value. The proteins are ordered according to the *p*-value. In the first column, the *p*-value varied from 5.25E–12 to 5.47E–05, in the second column from 5.97E–05 to 0.00283, and in the third column from 0.00284 to 0.04071. A value of *p* < 0.05 was considered statistically significant. Data were plotted using matrix2png version 1.2.2 (http://www.chibi.ubc.ca/matrix2png/). P1–P4 = Patient 1–Patient 4.

With the above-mentioned criteria, 651 proteins were quantified in all the patients before and after bariatric surgery (see Table S2). For optimum display and visualization, we used a Volcano plot that showed the log2 of the fold-change for each protein as a function of the *p*-value (Figure 1B). We considered proteins that significantly regulated those that have a *p*-value < 0.05 and a large fold-change > 1.5. After weight loss induced by bariatric surgery, in a statistically significant way, we found 228 proteins up-regulated and 136 down-regulated. The intensity changes of the differentially expressed proteins are shown as a heat map in Figure 2. 

To depict the functional classes significantly modulated in abdominal SAT after weight normalization induced by bariatric surgery, the two sets of differentially expressed proteins were functionally categorized using the FunRich software, which performs a hypergeometric test for the enrichment of GO terms [26].

**Figure 3:**
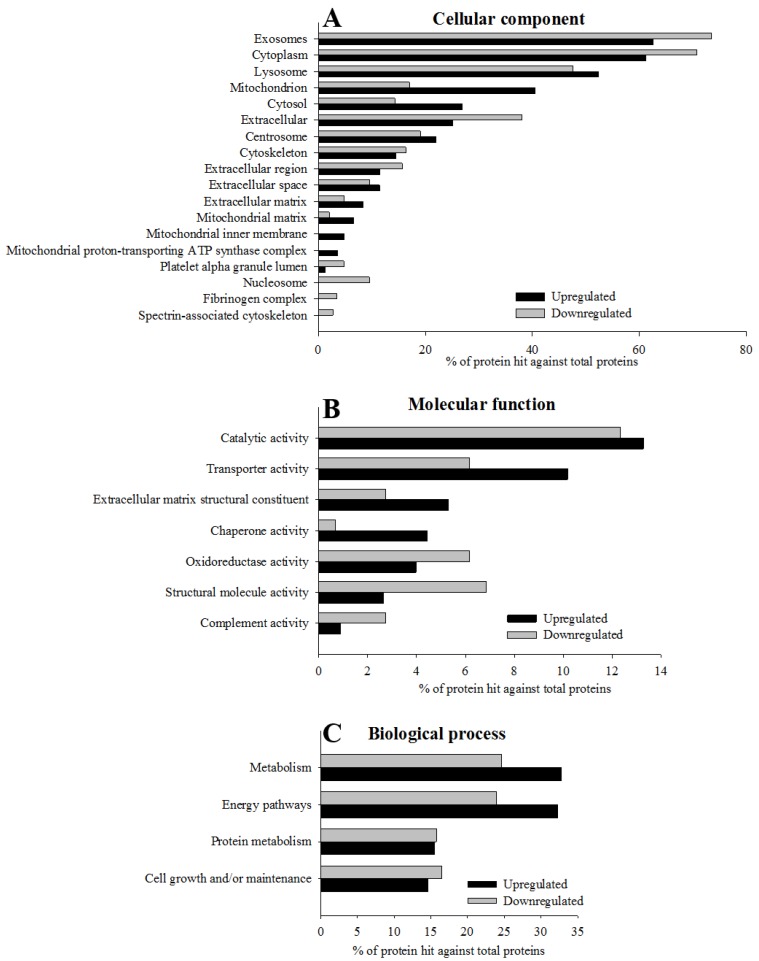
Gene ontology (GO) enrichment of 364 significantly regulated proteins according to the FunRich functional annotation. The histograms show for each GO term, cellular component (**A**; *p* < 10^−5^), biological process (**B**; *p* < 0.05) and molecular function (**C;** *p* < 0.05), the most significantly enriched categories of up-regulated and down-regulated proteins quantified using the SWATH-MS approach. Extended data of GO enrichment analysis is provided in Table S3.

Although in the GO enrichment analysis we found that the most significantly enriched GO cellular compartment for up and down-regulated proteins after weight loss were similar and were associated with exosomes, cytoplasm, lysosomes, mitochondria, cytosols, extracellular and centrosomes among others (see Figure 3A), we also detected differences between up- and down-regulated proteins. Up-regulated proteins after weight loss showed a higher enrichment of mitochondria as well as the mitochondrial matrix, mitochondrial inner membrane and mitochondrial proton-transportin ATP synthase complex in comparison with down-regulated proteins. On the contrary, down-regulated proteins were more enriched in the cellular component such as extracellular, nucleosome, and fibrinogen complex (see Figure 3A and Table S3 for the details of this analysis). For the molecular function, the major differences between up- and down-regulated proteins were related with transporter activity, extracellular matrix, structural constituent, chaperone activity (higher proportion on up-regulated proteins) and structural molecule activity and complement activity (higher enrichment on down-regulated proteins; see Figure 3B and Table S3 for the details of this analysis). If we observe the most enriched biological processes, these were related to the metabolic and energy pathways, and this was more evident in the up-regulated proteins after weight loss (see Figure 3C and Table S3 for the details of this analysis). 

In order to achieve a deeper understanding of the biological importance and significance of effects induced by BS and weight loss in aSAT, we performed functional reactome pathway enrichment analysis with the STRING tool (https://string-db.org/). Up-regulated proteins after bariatric surgery with the lowest *p*-value were associated with metabolism, the citric acid cycle and the respiratory electron transport, triglyceride catabolism and metabolism, formation of ATP, pyruvate metabolism, glycolysis/gluconeogenesis and thermogenesis among others (Table S4). In contrast, we found that the set of bariatric surgery down-regulated proteins with the lowest *p*-value was associated with pathways related to the immune system (Table S4). These results were comparable to those obtained in the qualitative analysis. 

Together, these results clearly demonstrated that bariatric surgery treatment induces alterations in metabolism, energy pathways and the immune system.

### 3.3. Validation of the SWATH-MS Analysis 

**Figure 4:**
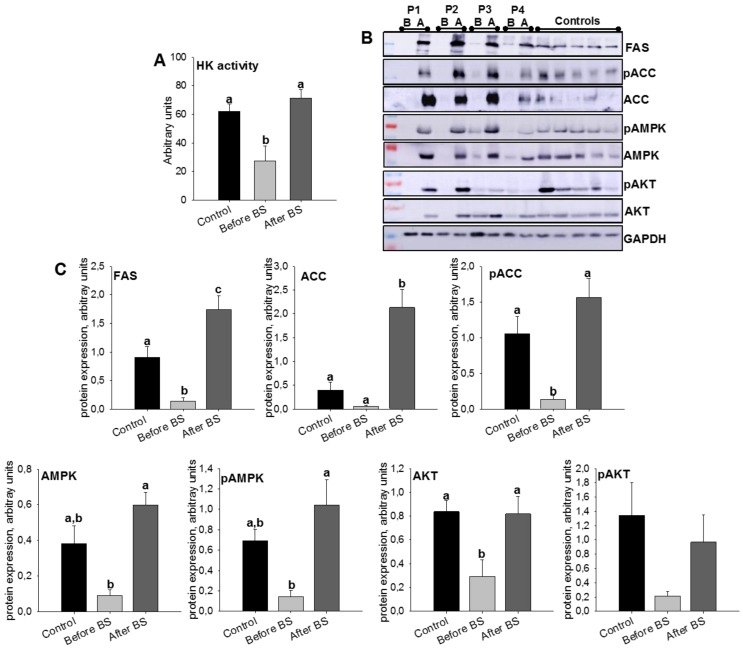
aSAT hexokinase (HK) activity levels (**A**) and protein expression levels (**B**,**C**) of lipid metabolism and insulin signaling-related enzymes, in control patients and obese patients before BS and the same patients after BS. Values are means ± SEM of six patients per group (HK activity) or 4–5 patients per group (Western blot). Different letters above the bars indicate statistically significant differences, *p* < 0.05. P.1–P.4 = Patient 1–Patient 4. B = before bariatric surgery, A = after bariatric surgery. Controls = healthy patients with normal BMI. BS = bariatric surgery.

In humans, it is known that increased lipolysis and impaired lipogenesis in AT lead to the release of cytokines and lipid metabolites, ultimately promoting insulin resistance [31]. After the analyzed proteins were quantified by SWATH-MS, we decided to study the effect of obesity and bariatric surgery on key enzymes of de novo lipogenesis and insulin signaling. To do so, we introduced a new group, a control, which constituted healthy patients with normal BMI. The results are shown in Figure 4A–C (Table S5 shows anthropometric and biochemical parameters for these patients). We began by studying the enzymatic activity of HK, since the phosphorylation of glucose limits metabolic processes such as glycolysis, and therefore de novo lipogenesis. Obesity clearly diminished HK activity, but the values were recovered by body weight normalization after BS. This same pattern was also observed in protein levels of FAS, ACC (total and phosphorylate), and AMPK (total and phosphorylate) confirming the results obtained for FAS in SWATH-MS quantification. However, for both FAS and ACC, we observed a significant fact, that weight loss after bariatric surgery not only restored their protein expression levels but they were even higher than those of the control patients, pointing to greater de novo lipogenesis. 

Insulin is a major endocrine hormone that is also involved in the regulation of energy and lipid metabolism. Under conditions of obesity, peripheral tissues experience a decrease in sensitivity to insulin [32]. Although there is controversy regarding the mechanism(s) that contribute to insulin resistance, AKT activation has been identified as a target [33]. Its activation leads to GLUT4 translocation into adipocyte membranes facilitating glucose uptake [33]. In Figure 4B,C, it can be observed that obesity decreased AKT protein levels (total and phosphorylate), levels that were restored after weight loss. 

Although all the data described so far are highly reproducible and coherent, in order to increase the strength of the results obtained by mass and subsequent bioinformatic analysis, we decided to go a step further and analyze the gene expression levels of *GLUT4* and proteins related to metabolism and inflammation that showed important changes in the SWATH-MS-analysis: *FAS*, *ADH1B* and *S100A8*. In this way, we were able to considerably increase the number of subjects both for control patients (*n* = 27) and for obese patients before weight loss (*n* = 145). We were unable to do the same for obese patients after bariatric surgery and weight loss, as it was very difficult to obtain these samples (*n* = 6), since many of the patients undergoing bariatric surgery, even if they lose weight, do not meet the requirements to undergo an abdominoplasty (Table S6 shows anthropometric and biochemical parameters for these patients).

**Figure 5:**
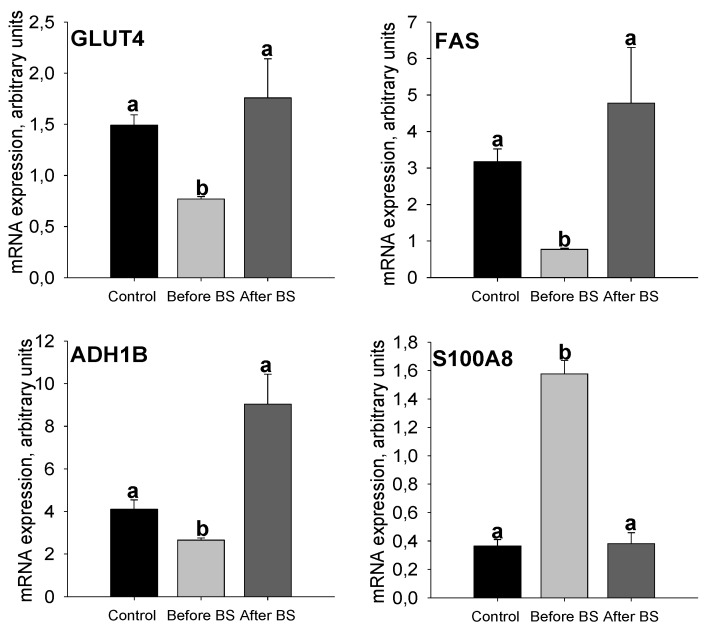
aSAT mRNA expression levels of *GLUT4*, *FAS*, *S100A8* and *ADH1B* in control patients and obese patients before BS and after BS and body weight normalization. Values are means ± SEM. Different letters above the bars indicate statistically significant differences, *p* < 0.05. Control = healthy patients with normal BMI, *n* = 27. Before BS, *n* = 145. After BS, *n* = 6. BS = bariatric surgery.

*GLUT4* mRNA expression levels decreased in the aSAT of obese patients, but these levels were recovered after weight loss (Figure 5). These results coincide with those seen for HK activity and AKT protein expression levels (Figure 4), suggesting that the entry of glucose into these cells is compromised during obesity, probably due to a decrease in the sensitivity of insulin, a problem that seems to be solved after the normalization of body weight. *FAS* and *ADH1B* mRNA expression levels (as it occurred with SWATH-MS analysis) decreased considerably during obesity and were restored after weight loss, even exceeding the values of the controls, although it was not statistically significant due to the reduced number of patients after BS (Figure 5). All these data seem to suggest that insulin sensitivity, as well as glucose uptake into the cell, glycolysis and de novo lipogenesis are decreased in aSAT during obesity but weight loss induced by bariatric surgery corrects these problems or even improves them with respect to controls.

S100A8 is a calcium- and zinc-binding protein that plays a prominent role in the regulation of inflammatory processes and immune responses including cardiovascular disease [34]. It is known that chronic low-grade inflammation of AT plays a crucial role in the pathophysiology of obesity [35] and increased expression of AT S100A8 can trigger macrophage mobility resulting in the progression of chronic inflammation in situ [36]. In the SWATH-MS analysis, the S100A8 protein showed a considerable decrease after weight loss induced by bariatric surgery. *S100A8* mRNA expression levels (Figure 5) are increased during obesity, but weight loss restores the values observed in the control patients group. In the same way as the data shown in Table S4, this suggests that weight loss induced by BS improves inflammation in aSAT.

**Figure 6:**
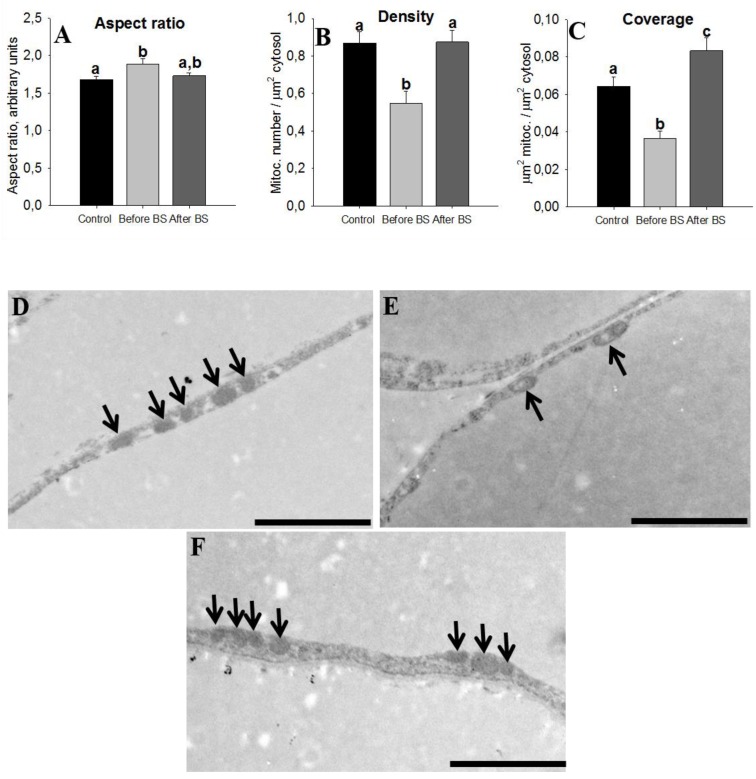
Mitochondrial morphometrics. Characterization of mitochondrial parameters in aSAT of control patients (healthy patients with normal BMI, *n* = 5), obese patients before bariatric surgery (before BS, *n* = 4), and obese patients after bariatric surgery and body weight normalization (after BS, *n* = 5). Values are means ± SEM. Different letters above the bars indicate statistically significant differences, *p* < 0.05. The aspect ratio is a measure of mitochondrial length (major axis/minor axis) (**A**), mitochondria density (**B**) and coverage (**C**) were calculated by dividing the number and total area of mitochondria to the cytoplasm area, respectively. Representative electron microscopy images of aSAT sections from control patients (**D**), obese patients before bariatric surgery (**E**) and obese patients after bariatric surgery (**F**), respectively. Bars = 2 µm. The arrows point to the mitochondria.

The AT of insulin-resistant humans displays a lower expression of proteins involved in mitochondrial function. In turn, this leads to a lower availability of mitochondria-derived energy sources for lipogenesis in AT [31]. We also have to take into account that many of the proteins that were positively regulated after weight loss were related to mitochondrial cellular components (see Figure 3A) and moreover that they were part of the pathways related to the respiratory electron transport and ATP synthesis (see Table S2). Our next step was to study mitochondrial morphometrics. Mitochondria shape (aspect ratio), density and coverage are shown in Figure 6. An aspect ratio (AR) value of 1 indicates a perfect circle, and as mitochondria elongate and become more elliptical, the AR increases. Therefore, it seems that obesity leads to slightly more elongated mitochondria, something that is corrected after weight loss but not in a statistically significant way (Figure 6A). Density and coverage diminished with obesity and weight loss recovered these values, even at higher levels than those observed in the controls (Figure 6B,C).

### 3.4. Adipocyte Size

**Figure 7:**
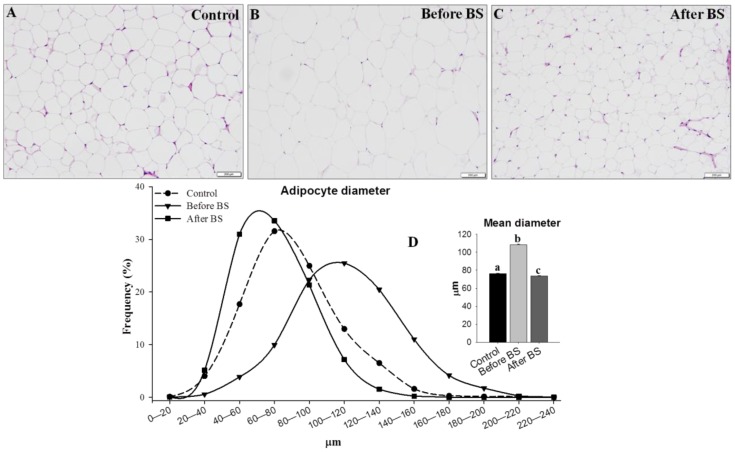
Adipocyte size distribution. The difference in adipocyte size is evident in representative H&E stained samples of aSAT from lean control patients (**A**, *n* = 14), obese patients before bariatric surgery (**B**, *n* = 14) and the same patients after bariatric surgery and normalization of body weight (**C**, *n* = 6). (**D**) The mean frequency of adipocyte diameter, indicating that obese patients before BS have greater numbers of adipocytes with diameters > 80 μm and fewer adipocytes with diameters < 80 μm when compared to control or obese patients after BS. Bars = 200 µm. Values are means ± SEM (histogram). Different letters above the bars indicate statistically significant differences, *p* < 0.05. BS = bariatric surgery.

Finally, we analyzed adipocyte diameters and distribution in control patients and the same obese patients before and after bariatric surgery and body weight normalization. To do so, we measured the diameter of more than 5000 adipocytes (1740 control, 1730 before BS and 1662 after BS). The results are shown in Figure 7. In all the groups studied, a normal distribution of adipocytes size was observed, with the size during obesity being greater than in the control and obese patients after body weight normalization. After weight loss, the mean size of the adipocytes was slightly lower than that observed in the control patients.

## 4. Discussion

We identified more than 1300 proteins and quantified 651 proteins, demonstrating that after normalization of the body weight, the proteome of aSAT had a much higher protein diversity. Moreover, 228 proteins were up-regulated and 136 were down-regulated after weight loss. Our results showed that up-regulated compared with down-regulated proteins after weight loss, showed higher enrichment for mitochondria as well as for the mitochondrial matrix, mitochondrial inner membrane and mitochondrial proton-transportin ATP synthase complex than down-regulated proteins. On the contrary, down-regulated proteins were more enriched in cellular components such as the extracellular, nucleosome and fibrinogen complex. When we grouped the altered proteins in the biological pathways, we observed that the pathways with the lowest *p*-value comprised of up-regulated proteins were associated with metabolism, the citric acid cycle and respiratory electron transport, triglyceride catabolism and metabolism, the formation of ATP, pyruvate metabolism, glycolysis/gluconeogenesis and thermogenesis among others. In contrast, proteins with decreased values after BS were part of a biological pathway related to the immune system. By using Western blot and RT-qPCR with additional samples in a control group with lean healthy volunteers, we confirmed that lipogenic enzymes were decreased with obesity but were corrected by BS, and even improved with respect the control group in the same cases. *GLUT4*, *FAS* and *ADH1B* mRNA expression levels showed a similar pattern. This state of “super-normality” was previously observed in women undergoing bariatric surgery, who at five years displayed more insulin sensitivity compared to controls [37].

The SAT is the largest and most adequate storage site for excess lipids. However, it has a limited ability to expand, and when it is inadequate, this leads to a hypertrophic expansion of the cells, reducing lipid storage and increasing lipolysis and the accumulation of ectopic fat promoting systemic insulin resistance. The limited expansion and storage capacity of SAT is a major cause of obesity-associated metabolic complications (reviewed by Hammarstedt et al. [38] and Gancheva et al. [39]. Accordingly, the increased lipid storage capacity of AT could prevent ectopic lipid accumulation, thereby preserving metabolic homeostasis and insulin resistance. All of these facts are consistent with our results. After weight loss is induced by BS, it is possible to observe an improvement in glucose uptake (higher HK activity) and de novo lipogenesis (represented by ACC and FAS protein and mRNA expression levels), at the same time that insulin sensitivity improves (homeostasis model assessment of insulin resistance (HOMA-IR) decreases as well as the number of patients medicated for T2D, at the same time that AKT levels increase). Consistent with all this, *GLUT4* mRNA expression levels, the main insulin-regulated glucose transporter, improved after BS. This could at least partly explain the increase in aSAT glucose uptake and de novo lipogenesis observed after BS. Moreover, it is known that reduced GLUT4 and lipogenesis in adipocytes impairs the synthesis of branched fatty acid esters of hydroxy fatty acids (FAHFAs) a family of lipids secreted by AT that enhance insulin-stimulated glucose transport and glucose-stimulated GLP1 and insulin secretion, and also have powerful anti-inflammatory effects (reviewed by Smith and Kahn [40]).

In humans, ADH1B is inversely correlated with waist circumference, BMI, fasting plasma insulin [41] and with fat cell size [42]. ADH1B has also been implicated in body weight regulation [43]. For all these reasons, it was suggested that ADH1B may be involved in AT expandability [42]. We also observed that ADH1B protein and mRNA expression levels decreased with obesity, but BS restored and even exceeded the values of the control group, demonstrating a negative correlation with BMI, insulin sensitivity and adipocytes size. Although this is not an irrefutable demonstration of the hypothesis presented by Voguel et al., it does support their theory, which means that after BS aSAT, expandability is improved with respect to the control group.

In response to excess energy, adipocytes undergo a remodeling that translates into hypertrophy and hyperplasia. It has been suggested that hypertrophy is associated with relative insulin sensitivity or early insulin resistance and hyperplasia with greater insulin resistance and T2D [44,45,46,47]. Since patients who have an extended population of small cells have impaired adipogenesis/fat storage, for this reason they have a higher risk of developing insulin resistance and T2D [48,49,50]. Some authors have noted that the distribution of adipocytes is bimodal or even trimodal [44,50,51]. However, we did not find any evidence of bimodal distribution during obesity in any of the tested conditions as observed by other authors [37]. In all of the groups, we observed a normal distribution of the adipocyte diameters with obese patients before BS who had a higher diameter. Body weight normalization by BS restored adipocyte diameter distribution. This reduction in adipocyte hypertrophy contributed to normal metabolic function since larger adipocytes were associated with T2D, metabolic disease and reduced capacity of AT to store energy or release free fatty acids [16]. 

Low-grade inflammation in AT is one of the main factors that contribute to the development of obesity-associated insulin resistance and cardiovascular disease [52,53]. Consistent with the obtained proteomic data showing evidence of the down-regulation of proteins related to the immune system after BS*, S100A8* mRNA expression levels increased with obesity and decreased after BS on aSAT. These results are in line with previous results obtained by other researchers. In humans, plasma S100A8/A9 levels have been correlated to insulin resistance [54] and obesity [54,55]. S100A8/A9 circulating levels high were related to a persistent diabetes status post-BS. Decreased levels of circulating S100A8/A9 were observed in diabetics patients before but not after BS and in nondiabetic patients, but not in diabetic patients before and after BS [56]. Moreover, reports on studies carried out with mice and humans suggest that S100A8 is involved in the recruitment of AT macrophages [36,57]. After weight loss, in addition to proteins related to the immune system, we also observed a decrease in proteins related to oxidative stress, such as peroxiredoxin-2 (PRDX2; *p*-value = 1.46^−10^), which in turn, has been suggested to link inflammation and oxidative stress as well as being related to the production of TNFα, an inflammatory cytokine [58].

Despite the well-established role in causing insulin resistance, the cause of AT inflammation is still not fully clear. AT hypoxia has been proposed as one of the possible hypotheses [59]. However, Woo et al. have recently proposed a new theory that mitochondrial dysfunction in adipocytes may be a primary cause of AT inflammation [60]. In previous studies, this group demonstrated that in white adipocytes, mitochondrial function is essential for adiponectin synthesis and that thiazolidinediones increase insulin sensitivity and adiponectin levels due to an increase in the mitochondrial content in adipocytes (reviewed by Woo et al. [60]). Inverse regulation of inflammation and mitochondrial function was found in a morbidly obese population where AT [61] and SAT of insulin-resistant humans and T2D patients displayed lower mitochondrial density [62,63]. In turn, this leads to lower availability of mitochondria-derived energy sources for lipogenesis. Abnormal mitochondrial function in human white AT likely contributes to the secretion of lipid metabolites and lactate, which are linked to insulin resistance in peripheral tissue [31]. In our study, we found multiple proteins related to the citric acid cycle, respiratory electron transport and formation of ATP by chemiosmotic coupling increased after bariatric surgery. Moreover, we demonstrated that obesity decreased the coverage and density of mitochondria and that bariatric surgery restored these values, demonstrating a relationship between mitochondrial dysfunction on aSAT and obesity. There is an inverse correlation between mitochondrial coverage/density and inflammation, and a direct correlation with glucose uptake, lipogenesis and insulin sensitivity. Tumor protein D54 (TPD54) is another protein that we found to be greatly increased after weight loss. Recently its mitochondrial location has been demonstrated, and it has been hypothesized that TPD54 knockdown decreases pyruvate dehydrogenase enzyme activity, reducing mitochondrial oxidative phosphorylation [64].

Our study has a number of strengths, but also some limitations: one of them is the low number of patients who were included in the study after losing weight. For this reason, we cannot rule out differences between sexes, the type of surgery performed or pathologies such as T2D. Moreover, we only analyzed the area corresponding to the adipose biopsy; therefore, we cannot rule out differences due to the depth at which the fat is located. However, the proteome was very homogeneous in all the patients analyzed. In addition, the genes and proteins analyzed, as well as the mitochondrial morphometry, confirm the results obtained by proteomic analysis, supporting the robustness of our study.

Future studies should analyze the evolution of these parameters over time, with a greater number of patients, to check which of them are dependent on weight loss and which improve before significant weight loss.

## 5. Conclusions

To the best of our knowledge, this work is the first “label-free” quantitative proteomics study using SWATH for the discovery of altered proteins with weight loss after bariatric surgery on aSAT. Over-represented pathways and protein groups are related to metabolism, the immune system, and mitochondrial function among others. In this work, we also showed specific molecular mechanisms, genes and proteins, that improve AT function characterized by lower inflammation, increased uptake glucose, higher insulin sensitivity, higher de novo lipogenesis, increased mitochondrial function and decreased adipocyte size. These data support that bariatric surgery remodels aSAT function contributing to the metabolic improvement that occurs both in AT and at the systemic level.

## Figures and Tables

**Table 1:** Anthropometric and biochemical parameters of patients used for mass spectrometric analysis.

	Patient 1		Patient 2		Patient 3		Patient 4	
**Gender**	**Female**		**Female**		**Female**		**Male**	
**Type of Bariatric Surgery**	**Sleeve**		**Sleeve **		**Bypass**		**Sleeve **	
	**Before BS**	**After BS**	**Before BS**	**After BS**	**Before BS**	**After BS**	**Before BS**	**After BS**
**Age** (years)	45.3	+27 m	31.4	+33 m	58	+24 m	45.4	+22 m
**D2M**	1	0	0	0	1	0	0	0
**Hypertension**	1	0	0	0	1	0	0	0
**BMI** (kg/m^2^)	49.2	29.2	44.6	26.3	39.8	29.2	66.3	29.8
**FAT** (%)	53	38.9	51.4	32.4	51.7	39.3	47.2	11.4
**Medication**	Amlodipine/Valsartan	Atorvastatin	0	0	Simvastatin Metformin Dapagliflozin	0	0	0
**Glucose** (mg/dL)	127	81	78	86	131	85	83	81
**Cholesterol** (mg/dL)	267	166	123	165	159	190	158	150
**HDLc** (mg/dL)	39	55	19	45	43	75	27	45
**LDLc** (mg/dL)	191	100	91	104	97	101	99	87
**Triglycerides** (mg/dL)	165	52	63	80	94	70	119	91
**AST** (UI/L)	27	13	21	14	61	76	28	22
**ALT** (UI/L)	36	9	18	10	51	99	70	20
**GGT** (UI/L)	68	19	17	14	40	37	96	35
**Insulin** (µUI/mL)	7.61	<2	3.3	3	11.4	3.1	8.1	<2
**ApoA** (mg/dL)	124	161	60.4	136	152	–	97.4	119
**ApoB** (mg/dL)	123	80.8	99.6	86.8	94.4	–	100	76.8
**CRP** (mg/dL)	2	0.32	0.88	0.02	1.5	–	1.33	0.01
**HbA1c** (%)	6.2	5	5.4	4.9	8	5.2	5.6	5.2
**HOMA-IR**	2.21	0.4	0.63	0.64	3.69	0.65	1.7	0.4

BMI: body mass index, HDLc: high-density lipoprotein cholesterol, LDLc: low-density lipoprotein cholesterol, AST: aspartate aminotransferase, ALT: alanine aminotransferase, GGT: γ-glutamyltransferase, ApoA: Apolipoprotein A, ApoB: Apolipoprotein B, HbA1c: Glycated hemoglobin, HOMA: index homeostasis model assessment index, CRP: C-reactive protein. BS = bariatric surgery. (–) data not available in the medical history.

## Supplementary Materials

The following are available online at https://www.mdpi.com/2077-0383/9/1/213/s1, Table S1: Enriched GO categories, protein class, and biological pathway within the differential body weight-expressed protein datasets, Table S2: Proteins quantified by SWATH-MS analysis in the aSAT, in the same patients before and after weight loss induced by bariatric surgery. BS = bariatric surgery. Proteins are sorted by *p*-value, Table S3: Significantly enriched GO categories (adj *p* < 0.05) within the differential weight loss regulated protein datasets. (FunRich database), Table S4: Reactome biological pathway enrichment analysis of statistically deregulated proteins by weight loss after bariatric surgery quantified using the SWATH-MS approach. A *p*-value < 10^−5^ was used as the cut-off criterion, Table S5: Anthropometric and biochemical parameters of patients used for Western blot analysis. Values are expressed as mean ± SEM. Different letters indicate statistical differences (*p* < 0.05 was considered statistically significant), Table S6: Anthropometric and biochemical parameters of patients used for quantitative real-time PCR analysis. Values are expressed as mean ± SEM. Different letters indicate statistical differences (*p* < 0.05 was considered statistically significant).

## Abbreviations

ACC: acetyl-CoA carboxylase; ADH1B: alcohol dehydrogenase 1B; AKT: serine/threonine protein kinase 1; ALT: alanine aminotransferase; AMPK: adenosine 5′ monophosphate-activated protein kinase; ApoA: Apolipoprotein A; ApoB: Apolipoprotein B; aSAT: abdominal subcutaneous adipose tissue; AST: aspartate aminotransferase; AT: adipose tissue; BMI: body mass index; BS: bariatric surgery; CRP: C-reactive protein; DDA: Data-Dependent Acquisition; FAHFAs: Branched fatty acid esters of hydroxy fatty acids; FAS: fatty acid synthase; FBXL10: F-box and leucine-rich repeat protein-10; FDR: false discovery rate; GAPDH: glyceraldehyde 3-phosphate dehydrogenase; GGT: γ-glutamyltransferase; GLUT4: glucose transporter type 4; GO: gene ontology; H&E: hematoxylin and eosin; HbA1c: Glycated hemoglobin; HDLc: high-density lipoprotein cholesterol; HK: Hexokinase; HOMA: index homeostasis model assessment index; IPO8: importin 8; LDLc: low-density lipoprotein cholesterol; MetS: metabolic syndrome; PCA: principal component analysis; PRDX2: peroxiredoxin-2; RYGB: Roux-en-Y gastric bypass; S100A8: S100 calcium binding protein A8; SAT: subcutaneous adipose tissue; SG: Sleeve Gastrectomy; SWATH-MS: Sequential Window Acquisition of all Theoretical Mass Spectra; T2D: type 2 diabetes; TOF: time-of-flight; TPD54: tumor protein D54; VAT: visceral adipose tissue.
