# Supplementary material for: Beneficial Effects of Bariatric Surgery-Induced by Weight Loss on the Proteome of Abdominal Subcutaneous Adipose Tissue
Source: J Clin Med. 2020 Jan 13;9(1):213. doi: 10.3390/jcm9010213 (PMC7019912; doi:10.3390/jcm9010213)
Supplement: Supplementary file 1 [file jcm-09-00213-s001.zip › jcm-666006-supplementary-corrections/Supplementary Table 1.docx]

| **MOLECULAR FUNCTION** | **OBESITY** | | **AFTER WEIGHT LOSS** | |  |
| --- | --- | --- | --- | --- | --- |
| **Category name (Accession)** | **# proteins** | **Percent of protein hit against total # proteins** | **# proteins** | **Percent of protein hit against total # proteins** | **Ratio Obesity/After weight loss** |
| Binding (GO:0005488) | 43 | 39.1% | 185 | 28.1% | 1.39 |
| Catalytic activity (GO:0003824) | 27 | 24.5% | 214 | 32.5% | 0.75 |
| Structural molecule activity (GO:0005198) | 7 | 6.4% | 44 | 6.7% | 0.96 |
| Transporter activity (GO:0005215) | 5 | 4.5% | 25 | 3.8% | 1.18 |
| Molecular transducer activity (GO:0060089) | 3 | 2.7% | 8 | 1.2% | 2.25 |
| Transcription regulator activity (GO:0140110) | 2 | 1.8% | 12 | 1.8% | 1.00 |
| Molecular function regulator (GO:0098772) | 2 | 1.8% | 14 | 2.1% | 0.86 |
| Translation regulator activity (GO:0045182) | 1 | 0.9% | 6 | 0.9% | 1.00 |

**Table S1.** Enriched GO categories, protein class, and biological pathway within the differential body weight-expressed protein datasets.

| **CELULAR COMPONENT** | **OBESITY** | | **AFTER WEIGHT LOSS** | |  |
| --- | --- | --- | --- | --- | --- |
| ***Category name (Accession)*** | ***# proteins*** | ***Percent of protein hit against total # proteins*** | ***# proteins*** | ***Percent of protein hit against total # proteins*** | ***Ratio Obesity/After weight loss*** |
| Cell (GO:0005623) | 33 | 30.0% | 227 | 34.5% | 0.87 |
| Extracellular region (GO:0005576) | 19 | 17.3% | 32 | 4.9% | 3.53 |
| Protein-containing complex (GO:0032991) | 15 | 13.6% | 61 | 9.3% | 1.46 |
| Membrane (GO:0016020) | 14 | 12.7% | 53 | 8.1% | 1.57 |
| Organelle (GO:0043226) | 10 | 9.1% | 101 | 15.3% | 0.59 |
| Cell junction (GO:0030054) | 5 | 4.5% | 4 | 0.6% | 7.5 |
| Supramolecular complex (GO:0099080) | 1 | 0.9% | 11 | 1.7% | 0.53 |

| **BIOLOGICAL PROCESS** | **OBESITY** | | **AFTER WEIGHT LOSS** | |  |
| --- | --- | --- | --- | --- | --- |
| ***Category name (Accession)*** | ***# proteins*** | ***Percent of protein hit against total # proteins*** | ***# proteins*** | ***Percent of protein hit against total # proteins*** | ***Ratio Obesity/After weight loss*** |
| Metabolic process (GO:0008152) | 30 | 27.3% | 229 | 34.8% | 0.78 |
| Biological regulation (GO:0065007) | 21 | 19.1% | 99 | 15.0% | 1.27 |
| Cellular component organization or biogenesis (GO:0071840) | 20 | 18.2% | 97 | 14.7% | 1.24 |
| Response to stimulus (GO:0050896) | 19 | 17.3% | 37 | 5.6% | 3.09 |
| Cellular process (GO:0009987) | 17 | 15.5% | 50 | 7.6% | 2.04 |
| Localization (GO:0051179) | 17 | 15.5% | 73 | 11.1% | 1.4 |
| Immune system process (GO:0002376) | 10 | 9.1% | 20 | 3.0% | 3.03 |
| Multicellular organismal process (GO:0032501) | 9 | 8.2% | 27 | 4.1% | 2.00 |
| Developmental process (GO:0032502) | 8 | 7.3% | 29 | 4.4% | 1.66 |
| Biological adhesion (GO:0022610) | 6 | 5.5% | 18 | 2.7% | 2.04 |
| Multi-organism process (GO:0051704) | 3 | 2.7% | 1 | 0.2% | 13.50 |
| Locomotion (GO:0040011) | 2 | 1.8% | - | - | - |
| Reproduction (GO:0000003) | 1 | 0.9% | 4 | 0.6% | 1.50 |
| Signalling (GO:0023052) | - | - | 7 | 1.1% | - |

| **PROTEIN CLASS** | **OBESITY** | | **AFTER WEIGHT LOSS** | |  |
| --- | --- | --- | --- | --- | --- |
| ***Category name (Accession)*** | ***# proteins*** | ***Percent of protein hit against total # proteins*** | ***# proteins*** | ***Percent of protein hit against total # proteins*** | ***Ratio Obesity/After weight loss*** |
| Cytoskeletal protein (PC00085) | 13 | 11.8% | 28 | 4.3% | 2.74 |
| Hydrolase (PC00121) | 12 | 10.9% | 65 | 9.9% | 1.10 |
| Nucleic acid binding (PC00171) | 7 | 6.4% | 75 | 11.4% | 0.56 |
| Oxidoreductase (PC00176) | 5 | 4.5% | 56 | 8.5% | 0.53 |
| Signalling molecule (PC00207) | 5 | 4.5% | 14 | 2.1% | 2.14 |
| Calcium-binding protein (PC00060) | 5 | 4.5% | 15 | 2.3% | 1.96 |
| Transporter (PC00227) | 4 | 3.6% | 15 | 2.3% | 1.57 |
| Transferase (PC00220) | 3 | 2.7% | 45 | 6.8% | 0.40 |
| Cell adhesion molecule (PC00069) | 2 | 1.8% | 5 | 0.8% | 2.25 |
| Ligase (PC00142) | 2 | 1.8% | 27 | 4.1% | 0.44 |
| Defense/immunity protein (PC00090) | 2 | 1.8% | 4 | 0.6% | 3.00 |
| Isomerase (PC00135) | 2 | 1.8% | 5 | 0.8% | 2.25 |
| Receptor (PC00197) | 2 | 1.8% | 12 | 1.8% | 1.00 |
| Extracellular matrix protein (PC00102) | 1 | 0.9% | 10 | 1.5% | 0.60 |
| Lyase (PC00144) | 1 | 0.9% | 9 | 1.4% | 0.64 |
| Enzyme modulator (PC00095) | 1 | 0.9% | 45 | 6.8% | 0.13 |
| Transfer/carrier protein (PC00219) | 1 | 0.9% | 14 | 2.1% | 0.43 |
| Transcription factor (PC00218) | 1 | 0.9% | 12 | 1.8% | 0.5 |
| Storage protein (PC00210) | 1 | 0.9% | 1 | 0.2% | 4.5 |
| Membrane traffic protein (PC00150) | - | - | 21 | 3.2% | - |
| Structural protein (PC00211) | - | - | 5 | 0.8% | - |
| Chaperone (PC00072) | - | - | 3 | 0.5% | - |
| Transmembrane receptor regulatory/adaptor protein (PC00226) | - | - | 2 | 0.3% | - |
| Cell junction protein (PC00070) | - | - | 1 | 0.2% | - |

| **PATHWAY. OBESITY** |  |  |
| --- | --- | --- |
| ***Category name (Accession)*** | ***# proteins*** | ***Percent of protein hit against total # proteins*** |
| Blood coagulation (P00011) | 6 | 5.5% |
| Wnt signaling pathway (P00057) | 5 | 4.5% |
| Integrin signalling pathway (P00034) | 4 | 3.6% |
| Inflammation mediated by chemokine and cytokine signaling pathway (P00031) | 4 | 3.6% |
| Cytoskeletal regulation by Rho GTPase (P00016) | 4 | 3.6% |
| Nicotinic acetylcholine receptor signaling pathway (P00044) | 3 | 2.7% |
| Cadherin signaling pathway (P00012) | 2 | 1.8% |
| Huntington disease (P00029) | 2 | 1.8% |
| De novo pyrimidine ribonucleotides biosythesis (P02740) | 1 | 0.9% |
| Pentose phosphate pathway (P02762) | 1 | 0.9% |
| Alzheimer disease-presenilin pathway (P00004) | 1 | 0.9% |
| Arginine biosynthesis (P02728) | 1 | 0.9% |
| Heterotrimeric G-protein signaling pathway-Gi alpha and Gs alpha mediated pathway (P00026) | 1 | 0.9% |
| Toll receptor signaling pathway (P00054) | 1 | 0.9% |
| Heme biosynthesis (P02746) | 1 | 0.9% |
| DNA replication (P00017) | 1 | 0.9% |

| **PATHWAY.** **AFTER WEIGHT LOSS** |  |  |
| --- | --- | --- |
| ***Category name (Accession)*** | ***# proteins*** | ***Percent of protein hit against total # proteins*** |
| Integrin signalling pathway (P00034) | 24 | 3.6% |
| Inflammation mediated by chemokine and cytokine signaling pathway (P00031) | 19 | 2.9% |
| Huntington disease (P00029) | 13 | 2.0% |
| CCKR signaling map (P06959) | 11 | 1.7% |
| Gonadotropin-releasing hormone receptor pathway (P06664) | 10 | 1.5% |
| Angiogenesis (P00005) | 9 | 1.4% |
| Ras Pathway (P04393) | 9 | 1.4% |
| Dopamine receptor mediated signaling pathway (P05912) | 9 | 1.4% |
| EGF receptor signaling pathway (P00018) | 9 | 1.4% |
| Ubiquitin proteasome pathway (P00060) | 8 | 1.2% |
| PDGF signaling pathway (P00047) | 8 | 1.2% |
| Heterotrimeric G-protein signaling pathway-Gi alpha and Gs alpha mediated pathway (P00026) | 8 | 1.2% |
| Endothelin signaling pathway (P00019) | 8 | 1.2% |
| Muscarinic acetylcholine receptor 2 and 4 signaling pathway (P00043) | 7 | 1.1% |
| p53 pathway feedback loops 2 (P04398) | 7 | 1.1% |
| FGF signaling pathway (P00021) | 7 | 1.1% |
| Wnt signaling pathway (P00057) | 6 | 0.9% |
| T cell activation (P00053) | 6 | 0.9% |
| Parkinson disease (P00049) | 6 | 0.9% |
| PI3 kinase pathway (P00048) | 6 | 0.9% |
| Metabotropic glutamate receptor group II pathway (P00040) | 6 | 0.9% |
| Interleukin signaling pathway (P00036) | 6 | 0.9% |
| Enkephalin release (P05913) | 6 | 0.9% |
| Cytoskeletal regulation by Rho GTPase (P00016) | 6 | 0.9% |
| Beta2 adrenergic receptor signaling pathway (P04378) | 6 | 0.9% |
| Beta1 adrenergic receptor signaling pathway (P04377) | 6 | 0.9% |
| 5HT1 type receptor mediated signaling pathway (P04373) | 6 | 0.9% |
| 5-Hydroxytryptamine degredation (P04372) | 6 | 0.9% |
| Apoptosis signaling pathway (P00006) | 5 | 0.8% |
| Toll receptor signaling pathway (P00054) | 5 | 0.8% |
| De novo purine biosynthesis (P02738) | 5 | 0.8% |
| Metabotropic glutamate receptor group III pathway (P00039) | 5 | 0.8% |
| Thyrotropin-releasing hormone receptor signaling pathway (P04394) | 5 | 0.8% |
| Oxytocin receptor mediated signaling pathway (P04391) | 5 | 0.8% |
| Histamine H2 receptor mediated signaling pathway (P04386) | 5 | 0.8% |
| Cortocotropin releasing factor receptor signaling pathway (P04380) | 5 | 0.8% |
| Cell cycle (P00013) | 5 | 0.8% |
| 5HT2 type receptor mediated signaling pathway (P04374) | 5 | 0.8% |
| Adrenaline and noradrenaline biosynthesis (P00001) | 4 | 0.6% |
| p53 pathway (P00059) | 4 | 0.6% |
| TCA cycle (P00051) | 4 | 0.6% |
| Muscarinic acetylcholine receptor 1 and 3 signaling pathway (P00042) | 4 | 0.6% |
| Metabotropic glutamate receptor group I pathway (P00041) | 4 | 0.6% |
| GABA-B receptor II signaling (P05731) | 4 | 0.6% |
| Interferon-gamma signaling pathway (P00035) | 4 | 0.6% |
| FAS signaling pathway (P00020) | 4 | 0.6% |
| Pyruvate metabolism (P02772) | 4 | 0.6% |
| Histamine H1 receptor mediated signaling pathway (P04385) | 4 | 0.6% |
| B cell activation (P00010) | 4 | 0.6% |
| Alzheimer disease-presenilin pathway (P00004) | 3 | 0.5% |
| Methylmalonyl pathway (P02755) | 3 | 0.5% |
| TGF-beta signaling pathway (P00052) | 3 | 0.5% |
| Nicotinic acetylcholine receptor signaling pathway (P00044) | 3 | 0.5% |
| JAK/STAT signaling pathway (P00038) | 3 | 0.5% |
| Heterotrimeric G-protein signaling pathway-rod outer segment phototransduction (P00028) | 3 | 0.5% |
| Heterotrimeric G-protein signaling pathway-Gq alpha and Go alpha mediated pathway (P00027) | 3 | 0.5% |
| Opioid proopiomelanocortin pathway (P05917) | 3 | 0.5% |
| Opioid prodynorphin pathway (P05916) | 3 | 0.5% |
| Opioid proenkephalin pathway (P05915) | 3 | 0.5% |
| Nicotine pharmacodynamics pathway (P06587) | 3 | 0.5% |
| Pyrimidine Metabolism (P02771) | 3 | 0.5% |
| Beta3 adrenergic receptor signaling pathway (P04379) | 3 | 0.5% |
| 5HT4 type receptor mediated signaling pathway (P04376) | 3 | 0.5% |
| Alzheimer disease-amyloid secretase pathway (P00003) | 2 | 0.3% |
| N-acetylglucosamine metabolism (P02756) | 2 | 0.3% |
| Alpha adrenergic receptor signaling pathway (P00002) | 2 | 0.3% |
| Heme biosynthesis (P02746) | 2 | 0.3% |
| VEGF signaling pathway (P00056) | 2 | 0.3% |
| Fructose galactose metabolism (P02744) | 2 | 0.3% |
| Oxidative stress response (P00046) | 2 | 0.3% |
| Acetate utilization (P02722) | 2 | 0.3% |
| Insulin/IGF pathway-protein kinase B signaling cascade (P00033) | 2 | 0.3% |
| ATP synthesis (P02721) | 2 | 0.3% |
| p53 pathway by glucose deprivation (P04397) | 2 | 0.3% |
| Glycolysis (P00024) | 2 | 0.3% |
| Succinate to proprionate conversion (P02777) | 2 | 0.3% |
| Angiotensin II-stimulated signaling through G proteins and beta-arrestin (P05911) | 2 | 0.3% |
| Cholesterol biosynthesis (P00014) | 2 | 0.3% |
| Blood coagulation (P00011) | 2 | 0.3% |
| Pentose phosphate pathway (P02762) | 2 | 0.3% |
| Axon guidance mediated by semaphorins (P00007) | 1 | 0.2% |
| O-antigen biosynthesis (P02757) | 1 | 0.2% |
| Methylcitrate cycle (P02754) | 1 | 0.2% |
| Leucine biosynthesis (P02749) | 1 | 0.2% |
| Isoleucine biosynthesis (P02748) | 1 | 0.2% |
| Glutamine glutamate conversion (P02745) | 1 | 0.2% |
| Transcription regulation by bZIP transcription factor (P00055) | 1 | 0.2% |
| Plasminogen activating cascade (P00050) | 1 | 0.2% |
| Endogenous cannabinoid signaling (P05730) | 1 | 0.2% |
| Aminobutyrate degradation (P02726) | 1 | 0.2% |
| Ionotropic glutamate receptor pathway (P00037) | 1 | 0.2% |
| Alanine biosynthesis (P02724) | 1 | 0.2% |
| Adenine and hypoxanthine salvage pathway (P02723) | 1 | 0.2% |
| Insulin/IGF pathway-mitogen activated protein kinase kinase/MAP kinase cascade (P00032) | 1 | 0.2% |
| Valine biosynthesis (P02785) | 1 | 0.2% |
| Hypoxia response via HIF activation (P00030) | 1 | 0.2% |
| Vitamin D metabolism and pathway (P04396) | 1 | 0.2% |
| Vasopressin synthesis (P04395) | 1 | 0.2% |
| p38 MAPK pathway (P05918) | 1 | 0.2% |
| Hedgehog signaling pathway (P00025) | 1 | 0.2% |
| Serine glycine biosynthesis (P02776) | 1 | 0.2% |
| Salvage pyrimidine ribonucleotides (P02775) | 1 | 0.2% |
| Gamma-aminobutyric acid synthesis (P04384) | 1 | 0.2% |
| Purine metabolism (P02769) | 1 | 0.2% |
| Cadherin signaling pathway (P00012) | 1 | 0.2% |
| 5HT3 type receptor mediated signaling pathway (P04375) | 1 | 0.2% |
