# Supplementary material for: Beneficial Effects of Bariatric Surgery-Induced by Weight Loss on the Proteome of Abdominal Subcutaneous Adipose Tissue
Source: J Clin Med. 2020 Jan 13;9(1):213. doi: 10.3390/jcm9010213 (PMC7019912; doi:10.3390/jcm9010213)
Supplement: Supplementary file 1 [file jcm-09-00213-s001.zip › jcm-666006-supplementary-corrections/Supplementary Table 3.docx]

**Table S3.** Significantly enriched GO categories (adjp < 0.05) within the differential weight loss regulated protein datasets. (FunRich database).

| **GO term** | **# of proteins in the background dataset** | **# of proteins in the dataset** | **% of proteins** | **Fold enrichment** | ***P*-value (Bonferroni method)** |
| --- | --- | --- | --- | --- | --- |
| *** Cellular Component** | | | | | |
| ***Enriched in down-regulated protein dataset*** | | | | | |
| *Exosomes* | 2043 | 108 | 73.47 | 5.23 | 1.31E-57 |
| *Lysosome* | 1620 | 70 | 47.62 | 4.28 | 6.77E-26 |
| *Nucleosome* | 44 | 14 | 9.52 | 31.51 | 4.26E-15 |
| *Extracellular* | 1825 | 56 | 38.10 | 3.04 | 1.89E-12 |
| *Cytoplasm* | 5684 | 104 | 70.75 | 1.81 | 4.16E-12 |
| *Cytoskeleton* | 427 | 24 | 16.33 | 5.57 | 5.66E-09 |
| *Centrosome* | 656 | 28 | 19.05 | 4.23 | 5.81E-08 |
| *Extracellular region* | 442 | 23 | 15.65 | 5.15 | 7.62E-08 |
| *Fibrinogen complex* | 7 | 5 | 3.40 | 70.75 | 1.59E-06 |
| *Platelet alpha granule lumen* | 35 | 7 | 4.76 | 19.82 | 3.80E-05 |
| *Spectrin-associated cytoskeleton* | 5 | 4 | 2.72 | 79.23 | 3.88E-05 |
| *Hemoglobin complex* | 10 | 4 | 2.72 | 39.66 | 1.56E-03 |
| *Platelet alpha granule* | 10 | 4 | 2.72 | 39.66 | 1.56E-03 |
| *Proteasome core complex* | 4 | 3 | 2.04 | 74.31 | 3.10E-03 |
| *Nucleoplasm* | 449 | 16 | 10.88 | 3.53 | 9.67E-03 |
| *Stored secretory granule* | 19 | 4 | 2.72 | 20.88 | 2.70E-02 |
| *Extracellular space* | 404 | 14 | 9.52 | 3.43 | 4.71E-02 |
| ***Enriched in up-regulated protein dataset*** | | | | | |
| *Exosomes* | 2043 | 142 | 62.56 | 4.46 | 4.64E-62 |
| *Lysosome* | 1620 | 119 | 52.42 | 4.71 | 3.43E-51 |
| *Mitochondrion* | 1259 | 92 | 40.53 | 4.69 | 1.37E-36 |
| *Centrosome* | 656 | 50 | 22.03 | 4.89 | 2.24E-18 |
| *Cytosol* | 1178 | 61 | 26.87 | 3.32 | 1.28E-14 |
| *Cytoskeleton* | 427 | 33 | 14.54 | 4.96 | 1.89E-11 |
| *Extracellular matrix* | 120 | 19 | 8.37 | 10.15 | 2.49E-11 |
| *Mitochondrial matrix* | 79 | 15 | 6.61 | 12.18 | 8.65E-10 |
| *Cytoplasm* | 5684 | 139 | 61.23 | 1.57 | 6.79E-09 |
| *Mitochondrial proton-transporting ATP synthase complex* | 18 | 8 | 3.52 | 28.51 | 9.29E-08 |
| *Extracellular space* | 404 | 26 | 11.45 | 4.13 | 7.24E-07 |
| *Mitochondrial inner membrane* | 58 | 11 | 4.85 | 12.17 | 9.79E-07 |
| *Extracellular region* | 442 | 26 | 11.45 | 3.77 | 4.80E-06 |
| *Extracellular* | 1825 | 57 | 25.11 | 2.00 | 1.17E-04 |
| *Nucleolus* | 1257 | 43 | 18.94 | 2.19 | 5.38E-04 |
| *Cytosolic small ribosomal subunit* | 36 | 7 | 3.08 | 12.48 | 9.12E-04 |
| *Mitochondrial proton-transporting ATP synthase. catalytic core* | 10 | 4 | 1.76 | 25.68 | 8.82E-03 |
| *Proteinaceous extracellular matrix* | 70 | 8 | 3.52 | 7.33 | 9.98E-03 |
| *Endoplasmic reticulum lumen* | 5 | 5 | 2.20 | 13.38 | 2.31E-02 |
| *Membrane* | 350 | 17 | 7.49 | 3.12 | 2.81E-02 |
| *Endoplasmic reticulum* | 35 | 35 | 15.42 | 2.03 | 3.45E-02 |
| *** Molecular function** | | | | | |
| ***Enriched in down-regulated protein dataset*** | | | | | |
| *Catalytic activity* | 532 | 18 | 12.33 | 4.20 | 6.46E-05 |
| *Oxidoreductase activity* | 161 | 9 | 6.16 | 6.95 | 1.41E-03 |
| *Structural molecule activity* | 269 | 10 | 6.85 | 4.62 | 1.48E-02 |
| *Complement activity* | 28 | 4 | 2.74 | 17.77 | 1.59E-02 |
| ***Enriched in up-regulated protein dataset*** | | | | | |
| *Catalytic activity* | 532 | 30 | 13.27 | 4.52 | 1.07E-09 |
| *Transporter activity* | 576 | 23 | 10.18 | 3.20 | 2.11E-04 |
| *Extracellular matrix structural constituent* | 166 | 12 | 5.31 | 5.80 | 2.66E-04 |
| *Chaperone activity* | 126 | 10 | 4.42 | 6.37 | 9.10E-04 |
| *Oxidoreductase activity* | 161 | 9 | 3.98 | 4.49 | 4.31E-02 |
| *** Biological process** | | | | | |
| ***Enriched in down-regulated protein dataset*** | | | | | |
| *Metabolism* | 1683 | 36 | 24.66 | 2.66 | 6.59E-06 |
| *Energy pathways* | 1633 | 35 | 23.97 | 2.66 | 1.02E-05 |
| *Cell growth and/or maintenance* | 1125 | 24 | 16.44 | 2.65 | 1.94E-03 |
| *Protein metabolism* | 1323 | 23 | 15.75 | 2.16 | 6.61E-02 |
| ***Enriched in up-regulated protein dataset*** | | | | | |
| *Energy pathways* | 1633 | 73 | 32.30 | 3.59 | 7.30E-21 |
| *Metabolism* | 1683 | 74 | 32.74 | 3.53 | 8.92E-21 |
| *Cell growth and/or maintenance* | 1125 | 33 | 14.60 | 2.35 | 7.09E-04 |
| *Protein metabolism* | 1323 | 35 | 15.49 | 2.12 | 3.31E-03 |
|  |  |  |  |  |  |
