# Supplementary material for: Beneficial Effects of Bariatric Surgery-Induced by Weight Loss on the Proteome of Abdominal Subcutaneous Adipose Tissue
Source: J Clin Med. 2020 Jan 13;9(1):213. doi: 10.3390/jcm9010213 (PMC7019912; doi:10.3390/jcm9010213)
Supplement: Supplementary file 1 [file jcm-09-00213-s001.zip › jcm-666006-supplementary-corrections/Supplementary table 4.docx]

**Table S4.** Reactome Biological Pathway enrichment analysis of statistically deregulated proteins by weight loss after bariatric surgery quantified using the SWATH-MS approach. A *p*-value < 10^−5^ was used as the cut-off criterion.

| ***Pathway identifier*** | ***Pathway name*** | ***# Entities found*** | ***# Entities total*** | ***Entities ratio*** | ***Entities p-value*** |
| --- | --- | --- | --- | --- | --- |
| ***Up-regulated*** | | | | | |
| *R-HSA-1430728* | Metabolism | 109 | 2132 | 0.191 | 1.11E-16 |
| *R-HSA-1428517* | The citric acid (TCA) cycle and respiratory electron transport | 24 | 175 | 0.016 | 7.94E-13 |
| *R-HSA-6798695* | Neutrophil degranulation | 36 | 480 | 0.043 | 5.06E-11 |
| *R-HSA-163560* | Triglyceride catabolism | 9 | 24 | 0.002 | 3.32E-09 |
| *R-HSA-163210* | Formation of ATP by chemiosmotic coupling | 8 | 18 | 0.002 | 6.84E-09 |
| *R-HSA-8979227* | Triglyceride metabolism | 9 | 38 | 0.003 | 1.61E-07 |
| *R-HSA-71406* | Pyruvate metabolism and Citric Acid (TCA) cycle | 10 | 55 | 0.005 | 3.65E-07 |
| *R-HSA-8949613* | Cristae formation | 8 | 31 | 0.003 | 4.19E-07 |
| *R-HSA-163200* | Respiratory electron transport; ATP synthesis by chemiosmotic coupling; and heat production by uncoupling proteins. | 14 | 124 | 0.011 | 5.44E-07 |
| *R-HSA-76002* | Platelet activation; signalling and aggregation | 20 | 262 | 0.024 | 9.95E-07 |
| *R-HSA-373755* | Semaphorin interactions | 10 | 64 | 0.006 | 1.41E-06 |
| *R-HSA-156842* | Eukaryotic Translation Elongation | 11 | 95 | 0.009 | 7.19E-06 |
| *R-HSA-71387* | Metabolism of carbohydrates | 20 | 300 | 0.027 | 7.39E-06 |
| *R-HSA-71403* | Citric acid cycle (TCA cycle) | 6 | 22 | 0.002 | 8.82E-06 |
| *R-HSA-70263* | Gluconeogenesis | 7 | 34 | 0.003 | 9.62E-06 |
| ***Down-regulated*** | | | | | |
| *R-HSA-168249* | Innate Immune System | 49 | 1324 | 0.094 | 5.12E-13 |
| *R-HSA-114608* | Platelet degranulation | 16 | 137 | 0.010 | 1.63E-11 |
| *R-HSA-76005* | Response to elevated platelet cytosolic Ca^2+^ | 16 | 144 | 0.010 | 3.37E-11 |
| *R-HSA-168256* | Immune System | 67 | 2662 | 0.188 | 3.52E-10 |
| *R-HSA-8941858* | Regulation of RUNX3 expression and activity | 10 | 57 | 0.004 | 2.65E-09 |
| *R-HSA-211733* | Regulation of activated PAK-2p34 by proteasome-mediated degradation | 9 | 50 | 0.004 | 1.35E-08 |
| *R-HSA-180534* | Vpu mediated degradation of CD4 | 9 | 53 | 0.004 | 2.22E-08 |
| *R-HSA-69601* | Ubiquitin Mediated Degradation of Phosphorylated Cdc25A | 9 | 54 | 0.004 | 2.60E-08 |
| *R-HSA-69613* | p53-Independent G1/S DNA damage checkpoint | 9 | 54 | 0.004 | 2.60E-08 |
| *R-HSA-69610* | p53-Independent DNA Damage Response | 9 | 54 | 0.004 | 2.60E-08 |
| *R-HSA-75815* | Ubiquitin-dependent degradation of Cyclin D | 9 | 54 | 0.004 | 2.60E-08 |
| *R-HSA-169911* | Regulation of Apoptosis | 9 | 54 | 0.004 | 2.60E-08 |
| *R-HSA-349425* | Autodegradation of the E3 ubiquitin ligase COP1 | 9 | 54 | 0.004 | 2.60E-08 |
| *R-HSA-8854050* | FBXL7 down-regulates AURKA during mitotic entry and in early mitosis | 9 | 55 | 0.004 | 3.03E-08 |
| *R-HSA-174113* | SCF-beta-TrCP mediated degradation of Emi1 | 9 | 55 | 0.004 | 3.03E-08 |
| *R-HSA-180585* | Vif-mediated degradation of APOBEC3G | 9 | 56 | 0.004 | 3.53E-08 |
| *R-HSA-450408* | AUF1 (hnRNP D0) binds and destabilizes mRNA | 9 | 56 | 0.004 | 3.53E-08 |
| *R-HSA-4641258* | Degradation of DVL | 9 | 57 | 0.004 | 4.10E-08 |
| *R-HSA-4641257* | Degradation of AXIN | 9 | 57 | 0.004 | 4.10E-08 |
| *R-HSA-9604323* | Negative regulation of NOTCH4 signalling | 9 | 57 | 0.004 | 4.10E-08 |
| *R-HSA-5673001* | RAF/MAP kinase cascade | 17 | 274 | 0.019 | 4.61E-08 |
| *R-HSA-1247673* | Erythrocytes take up oxygen and release carbon dioxide | 6 | 16 | 0.001 | 5.46E-08 |
| *R-HSA-68827* | CDT1 association with the CDC6:ORC:origin complex | 9 | 59 | 0.004 | 5.47E-08 |
| *R-HSA-69541* | Stabilization of p53 | 9 | 59 | 0.004 | 5.47E-08 |
| *R-HSA-5684996* | MAPK1/MAPK3 signalling | 17 | 281 | 0.020 | 6.58E-08 |
| *R-HSA-5362768* | Hh mutants that don't undergo autocatalytic processing are degraded by ERAD | 9 | 61 | 0.004 | 7.24E-08 |
| *R-HSA-5676590* | NIK-->noncanonical NF-kB signalling | 9 | 61 | 0.004 | 7.24E-08 |
| *R-HSA-5610780* | Degradation of GLI1 by the proteasome | 9 | 62 | 0.004 | 8.29E-08 |
| *R-HSA-5610785* | GLI3 is processed to GLI3R by the proteasome | 9 | 62 | 0.004 | 8.29E-08 |
| *R-HSA-5610783* | Degradation of GLI2 by the proteasome | 9 | 62 | 0.004 | 8.29E-08 |
| *R-HSA-187577* | SCF(Skp2)-mediated degradation of p27/p21 | 9 | 62 | 0.004 | 8.29E-08 |
| *R-HSA-8939236* | RUNX1 regulates transcription of genes involved in differentiation of HSCs | 11 | 106 | 0.007 | 8.61E-08 |
| *R-HSA-6798695* | Neutrophil degranulation | 22 | 480 | 0.034 | 9.16E-08 |
| *R-HSA-174084* | Autodegradation of Cdh1 by Cdh1:APC/C | 9 | 63 | 0.004 | 9.48E-08 |
| *R-HSA-5387390* | Hh mutants abrogate ligand secretion | 9 | 64 | 0.005 | 1.08E-07 |
| *R-HSA-4608870* | Asymmetric localization of PCP proteins | 9 | 66 | 0.005 | 1.40E-07 |
| *R-HSA-5607761* | Dectin-1 mediated noncanonical NF-kB signalling | 9 | 66 | 0.005 | 1.40E-07 |
| *R-HSA-174154* | APC/C:Cdc20 mediated degradation of Securin | 9 | 67 | 0.005 | 1.58E-07 |
| *R-HSA-5678895* | Defective CFTR causes cystic fibrosis | 9 | 67 | 0.005 | 1.58E-07 |
| *R-HSA-68867* | Assembly of the pre-replicative complex | 9 | 68 | 0.005 | 1.79E-07 |
| *R-HSA-354194* | GRB2:SOS provides linkage to MAPK signalling for Integrins | 6 | 20 | 0.001 | 2.00E-07 |
| *R-HSA-109582* | Hemostasis | 29 | 826 | 0.058 | 2.07E-07 |
| *R-HSA-5658442* | Regulation of RAS by GAPs | 9 | 70 | 0.005 | 2.28E-07 |
| *R-HSA-1234176* | Oxygen-dependent proline hydroxylation of Hypoxia-inducible Factor Alpha | 9 | 70 | 0.005 | 2.28E-07 |
| *R-HSA-69580* | p53-Dependent G1/S DNA damage checkpoint | 9 | 70 | 0.005 | 2.28E-07 |
| *R-HSA-69563* | p53-Dependent G1 DNA Damage Response | 9 | 70 | 0.005 | 2.28E-07 |
| *R-HSA-350562* | Regulation of ornithine decarboxylase (ODC) | 8 | 52 | 0.004 | 2.86E-07 |
| *R-HSA-174178* | APC/C:Cdh1 mediated degradation of Cdc20 and other APC/C:Cdh1 targeted proteins in late mitosis/early G1 | 9 | 72 | 0.005 | 2.88E-07 |
| *R-HSA-174184* | Cdc20:Phospho-APC/C mediated degradation of Cyclin A | 9 | 72 | 0.005 | 2.88E-07 |
| *R-HSA-69615* | G1/S DNA Damage Checkpoints | 9 | 72 | 0.005 | 2.88E-07 |
| *R-HSA-1169091* | Activation of NF-kappaB in B cells | 9 | 72 | 0.005 | 2.88E-07 |
| *R-HSA-5358346* | Hedgehog ligand biogenesis | 9 | 72 | 0.005 | 2.88E-07 |
| *R-HSA-179419* | APC:Cdc20 mediated degradation of cell cycle proteins prior to satisfaction of the cell cycle checkpoint | 9 | 73 | 0.005 | 3.22E-07 |
| *R-HSA-68949* | Orc1 removal from chromatin | 9 | 73 | 0.005 | 3.22E-07 |
| *R-HSA-372708* | p130Cas linkage to MAPK signalling for integrins | 6 | 22 | 0.002 | 3.48E-07 |
| *R-HSA-69017* | CDK-mediated phosphorylation and removal of Cdc6 | 9 | 74 | 0.005 | 3.61E-07 |
| *R-HSA-8948751* | Regulation of PTEN stability and activity | 9 | 74 | 0.005 | 3.61E-07 |
| *R-HSA-5689603* | UCH proteinases | 10 | 98 | 0.007 | 3.93E-07 |
| *R-HSA-176409* | APC/C:Cdc20 mediated degradation of mitotic proteins | 9 | 75 | 0.005 | 4.03E-07 |
| *R-HSA-1237044* | Erythrocytes take up carbon dioxide and release oxygen | 6 | 23 | 0.002 | 4.49E-07 |
| *R-HSA-1480926* | O2/CO2 exchange in erythrocytes | 6 | 23 | 0.002 | 4.49E-07 |
| *R-HSA-176814* | Activation of APC/C and APC/C:Cdc20 mediated degradation of mitotic proteins | 9 | 76 | 0.005 | 4.49E-07 |
| *R-HSA-5683057* | MAPK family signalling cascades | 17 | 332 | 0.023 | 6.63E-07 |
| *R-HSA-8878171* | Transcriptional regulation by RUNX1 | 15 | 261 | 0.018 | 7.64E-07 |
| *R-HSA-76002* | Platelet activation; signalling and aggregation | 16 | 299 | 0.021 | 8.16E-07 |
| *R-HSA-176408* | Regulation of APC/C activators between G1/S and early anaphase | 9 | 82 | 0.006 | 8.38E-07 |
| *R-HSA-449147* | Signalling by Interleukins | 24 | 641 | 0.045 | 8.72E-07 |
| *R-HSA-8939902* | Regulation of RUNX2 expression and activity | 9 | 83 | 0.006 | 9.25E-07 |
| *R-HSA-1234174* | Cellular response to hypoxia | 9 | 83 | 0.006 | 9.25E-07 |
| *R-HSA-8852276* | The role of GTSE1 in G2/M progression after G2 checkpoint | 9 | 83 | 0.006 | 9.25E-07 |
| *R-HSA-9020702* | Interleukin-1 signalling | 10 | 109 | 0.008 | 1.02E-06 |
| *R-HSA-977606* | Regulation of Complement cascade | 11 | 139 | 0.010 | 1.21E-06 |
| *R-HSA-69202* | Cyclin E associated events during G1/S transition | 9 | 88 | 0.006 | 1.49E-06 |
| *R-HSA-69002* | DNA Replication Pre-Initiation | 9 | 88 | 0.006 | 1.49E-06 |
| *R-HSA-453276* | Regulation of mitotic cell cycle | 9 | 90 | 0.006 | 1.78E-06 |
| *R-HSA-174143* | APC/C-mediated degradation of cell cycle proteins | 9 | 90 | 0.006 | 1.78E-06 |
| *R-HSA-195253* | Degradation of beta-catenin by the destruction complex | 9 | 90 | 0.006 | 1.78E-06 |
| *R-HSA-69656* | Cyclin A:Cdk2-associated events at S phase entry | 9 | 90 | 0.006 | 1.78E-06 |
| *R-HSA-8878159* | Transcriptional regulation by RUNX3 | 10 | 118 | 0.008 | 2.05E-06 |
| *R-HSA-69052* | Switching of origins to a post-replicative state | 9 | 92 | 0.006 | 2.13E-06 |
| *R-HSA-1168372* | Downstream signalling events of B Cell Receptor (BCR) | 9 | 92 | 0.006 | 2.13E-06 |
| *R-HSA-5632684* | Hedgehog 'on' state | 9 | 92 | 0.006 | 2.13E-06 |
| *R-HSA-9013694* | Signalling by NOTCH4 | 9 | 92 | 0.006 | 2.13E-06 |
| *R-HSA-450531* | Regulation of mRNA stability by proteins that bind AU-rich elements | 9 | 93 | 0.007 | 2.32E-06 |
| *R-HSA-5686938* | Regulation of TLR by endogenous ligand | 6 | 31 | 0.002 | 2.49E-06 |
| *R-HSA-162909* | Host Interactions of HIV factors | 11 | 150 | 0.011 | 2.50E-06 |
| *R-HSA-8950505* | Gene and protein expression by JAK-STAT signalling after Interleukin-12 stimulation | 8 | 73 | 0.005 | 3.49E-06 |
| *R-HSA-166658* | Complement cascade | 11 | 156 | 0.011 | 3.61E-06 |
| *R-HSA-5619084* | ABC transporter disorders | 9 | 99 | 0.007 | 3.84E-06 |
| *R-HSA-4086400* | PCP/CE pathway | 9 | 99 | 0.007 | 3.84E-06 |
| *R-HSA-1236978* | Cross-presentation of soluble exogenous antigens (endosomes) | 7 | 53 | 0.004 | 4.35E-06 |
| *R-HSA-5668541* | TNFR2 noncanonical NF-kB pathway | 9 | 104 | 0.007 | 5.68E-06 |
| *R-HSA-5687128* | MAPK6/MAPK4 signalling | 9 | 106 | 0.007 | 6.61E-06 |
| *R-HSA-354192* | Integrin alphaIIb beta3 signalling | 6 | 39 | 0.003 | 9.12E-06 |
| *R-HSA-9006921* | Integrin signalling | 6 | 39 | 0.003 | 9.12E-06 |
| *R-HSA-9020591* | Interleukin-12 signalling | 8 | 84 | 0.006 | 9.60E-06 |
