# Supplementary material for: Beneficial Effects of Bariatric Surgery-Induced by Weight Loss on the Proteome of Abdominal Subcutaneous Adipose Tissue
Source: J Clin Med. 2020 Jan 13;9(1):213. doi: 10.3390/jcm9010213 (PMC7019912; doi:10.3390/jcm9010213)
Supplement: Supplementary file 1 [file jcm-09-00213-s001.zip › jcm-666006-supplementary-corrections/Supplementary Table 5 + Supplementary Table 6.docx]

**Table S5. Anthropometric and biochemical parameters of patients used for Western blot analysis.** Values are expressed as mean ± SEM. Different letters indicate statistical differences (*p* < 0.05 was considered statistically significant).

|  | ***Control (n = 5)*** | ***Before bariatric surgery (n = 4)*** | ***After bariatric Surgery (n = 4)*** |
| --- | --- | --- | --- |
| ***Gender*** | 3 ♂ / 2 ♀ | 1 ♂ / 3♀ | 1 ♂ / 3♀ |
| ***Age (years)*** | 45.78 ± 4.36 | 45.04 ± 5.43 | 47.26 ± 5.26 |
| ***BMI (kg/m^2^)*** | 24.23 ± 0.45 a | 49.98 ± 5.77 b | 28.63 ± 0.79 ab |
| ***Glucose (mg/dl)*** | 90.40 ± 2.80 | 104.75 ± 14.06 | 83.25 ± 1.32 |
| ***Cholesterol (mg/dl)*** | 188.67 ± 33.75 | 176.75 ± 31.23 | 167.75 ± 8.27 |
| ***HDLc (mg/dl)*** | 63.50 ± 0.50 | 32.00 ± 5.51 | 55.00 ± 7.07 |
| ***LDLc (mg/dl)*** | 128.90 ± 24.90 | 119.50 ± 23.89 | 98.00 ± 3.76 |
| ***Triglycerides (mg/dl)*** | 135.00 ± 35.50 | 110.25 ± 21.55 | 73.25 ± 8.28 |
| ***AST (UI/L)*** | 25.50 ± 4.84 | 34.25 ± 9.05 | 31.25 ± 15.05 |
| ***ALT (UI/L)*** | 42.50 ± 11.55 | 43.75 ± 11.05 | 34.50 ± 21.64 |
| ***GGT (UI/L)*** | 47.67 ± 17.80 | 55.25 ± 17.12 | 26.25 ± 5.74 |
| ***Insulin (µUI/ml)*** | **-** | 7.60 ± 1.66 a | 2.53 ± 0.30 b |
| ***ApoA (mg/dl)*** | **-** | 108.45 ± 19.51 | 138.67 ± 12.20 |
| ***ApoB (mg/dl)*** | **-** | 104.25 ± 6.38 a | 81.47 ± 2.91 b |
| ***CRP (mg/dl)*** | **-** | 1.43 ± 0.23 a | 0.12 ± 0.10 b |
| ***HbA1c (%)*** | **-** | 6.30 ± 0.59 | 5.08 ± 0.08 |
| ***HOMA-IR*** | **-** | 2.06 ± 0.64 | 0.52 ± 0.07 (p=0.053) |
| ***Antidiabetic drugs*** | 0 % | 25.00 % | 0 % |
| ***Anti-lipid drugs*** | 0 % | 25.00 % | 0 % |

**Table S6. Anthropometric and biochemical parameters of patients used for quantitative real-time PCR analysis.** Values are expressed as mean ± SEM. Different letters indicate statistical differences (*p* < 0.05 was considered statistically significant).

|  | ***Control (n = 27)*** | ***Before bariatric surgery (n = 145)*** | ***After bariatric Surgery (n = 6)*** |
| --- | --- | --- | --- |
| ***Gender*** | 21♂ / 6 ♀ | 35♂ / 110 ♀ | 2♂ / 4♀ |
| ***Age (years)*** | 47.63 ± 2.14 | 46.84 ± 0.78 | 53.13 ± 4.98 |
| ***BMI (kg/m^2^)*** | 24.51 ± 0.28 a | 49.54 ± 0.70 b | 29.90 ± 0.95 a |
| ***Glucose (mg/dl)*** | 86.07 ± 1.30 | 104.55 ± 2.26 | 85.50 ± 2.85 |
| ***Cholesterol (mg/dl)*** | 203.75 ± 16.34 a | 140.31 ± 2.75 b | 173.17 ± 6.67 a |
| ***HDLc (mg/dl)*** | 61.86 ± 1.77 a | 30.33 ± 0.57 b | 52.80 ± 5.90 a |
| ***LDLc (mg/dl)*** | 131.57 ± 14.24 a | 86.13 ± 2.39 b | 102.20 ± 5.11 ab |
| ***Triglycerides (mg/dl)*** | 110.13 ± 14.70 | 121.28± 4.72 | 89.17± 13.46 |
| ***AST (UI/L)*** | 24.56 ± 2.99 a | 43.23 ± 2.29 b | 31.80 ± 11.67 ab |
| ***ALT (UI/L)*** | 31.90 ± 5.85 | 49.29 ± 2.79 | 32.50 ± 14.25 |
| ***GGT (UI/L)*** | 28.35 ± 4.43 | 37.07 ± 5.42 | 23.20 ± 5.39 |
| ***Insulin (µUI/ml)*** | - | 8.57 ± 0.79 a | 2.42 ± 0.26 b |
| ***ApoA (mg/dl)*** | - | 112.61 ± 2.08 a | 137.75 ± 8.67 b |
| ***ApoB (mg/dl)*** | - | 87.63 ± 2.29 | 81.93 ± 2.11 |
| ***CRP (mg/dl)*** | - | 0.93 ± 0.07 a | 0.09 ± 0.08 b |
| ***HbA1c (%)*** | - | 5.59 ± 0.08 | 5.16 ± 0.10 |
| ***HOMA-IR*** | - | 2.44 ± 0.30 a | 0.50 ± 0.06 b |
| ***Antidiabetic drugs*** | 0 % | 28.28 % | 0 % |
| ***Anti-lipid drugs*** | 0 % | 32.41 % | 16.67 % |
